# Assessment of transcriptional reprogramming of lettuce roots in response to chitin soil amendment

**DOI:** 10.3389/fpls.2023.1158068

**Published:** 2023-04-05

**Authors:** Leilei Li, Moritz Kaufmann, Moffat Makechemu, Christof Van Poucke, Ellen De Keyser, Mieke Uyttendaele, Cyril Zipfel, Bart Cottyn, Joël F. Pothier

**Affiliations:** ^1^ Plant Sciences Unit, Flanders Research Institute for Agriculture, Fisheries and Food (ILVO), Merelbeke, Belgium; ^2^ Department of Food Technology, Safety and Health, Ghent University, Ghent, Belgium; ^3^ Environmental Genomics and Systems Biology Research Group, Institute of Natural Resource Sciences, Zurich University of Applied Sciences (ZHAW), Wädenswil, Switzerland; ^4^ Department of Plant and Microbial Biology, Zurich-Basel Plant Science Center, University of Zurich, Zurich, Switzerland; ^5^ Technology and Food Science Unit, ILVO, Melle, Belgium; ^6^ The Sainsbury Laboratory, University of East Anglia, Norwich Research Park, Norwich, United Kingdom

**Keywords:** chitin, lettuce, RNA-Seq, phenolic compounds, plant defense

## Abstract

Chitin soil amendment is known to improve soil quality, plant growth and stress resilience, but the underlying mechanisms are not well understood. In this study, we monitored chitin’s effect on lettuce physiology every two weeks through an eight-week growth period, analyzed the early transcriptional reprogramming and related metabolomic changes of lettuce, in response to crab chitin treatment in peat-based potting soil. In commercial growth conditions, chitin amendment still promoted lettuce growth, increased chlorophyll content, the number of leaves and crop head weight from week six. The flavonoid content in lettuce leaves was altered as well, showing an increase at week two but a decrease from week six. Transcriptomic analysis showed that over 300 genes in lettuce root were significantly differentially expressed after chitin soil treatment. Gene Ontology-term (GO) enrichment analysis revealed statistical overrepresentation of GO terms linked to photosynthesis, pigment metabolic process and phenylpropanoid metabolic process. Further analysis of the differentially expressed genes (DEGs) showed that the flavonoid pathway was mostly upregulated whereas the bifurcation of upstream phenylpropanoid pathway towards lignin biosynthesis was mostly downregulated. Metabolomic analysis revealed the upregulation of salicylic acid, chlorogenic acid, ferulic acid, and *p*-coumaric acid in chitin-treated lettuce seedlings. These phenolic compounds (PCs) mainly influence the phenylpropanoid biosynthesis pathway and may play important roles in plant defense reactions. Our results suggest that chitin soil amendments might activate induced resistance by priming lettuce plants and promote lettuce growth *via* transcriptional changes.

## 1 Introduction

Lettuce (*Lactuca sativa* L.) is an economic important leafy vegetable cultivated in many countries around the world on a total area of < 1.8 M ha in 2021 (FAOSTAT, 2021). Lettuce constitutes an important source of vitamins, carotenoids and antioxidants (Mou, 2008). Chemical fertilizers and pesticides are commonly employed in lettuce cultivation to achieve higher crop yields (Subbarao et al., 2017). However, the use of chemical fertilizers and pesticides can lead to environmental pollution and affect human and animal health (Savci, 2012). Therefore, environmental-friendly alternatives for synthetic fertilizer and pesticides are recommended for sustainable agriculture (Kumar, 2012; Chen et al., 2018).

Chitin has drawn much attention in the past few decades, not only for its use as environmental-friendly fertilizer, but also because of its plant defense-promoting effect on various plants (Muymas et al., 2015; Debode et al., 2016; Shamshina et al., 2020; De Tender et al., 2021). Chitin is the second most abundant polysaccharide on earth, after cellulose. It is found in various organisms, including the exoskeletons of arthropods, cell walls of fungi, and the spines of diatoms (Sharp, 2013). Chitin acts as a fertilizer in soil by biodegrading into ammonia, which also supports the growth of specific microorganisms (Dahiya et al., 2006). Biodegradation is achieved by bacterial chitinases, enzymes that degrade chitin: endochitinases, exochitinases, and β-*N*-acetylhexosaminidases (Andronopoulou and Vorgias, 2004). Endochitinases break down the polymer chain by hydrolyzing random bonds to produce oligomers, which are further degraded. Exochitinase, on the other hand, releases diacetylchitobiose units at the polymer ends, while β-*N*-acetylhexosaminidases produce *N*-acetylglucosamine monomers from oligomers (Velásquez and Pirela, 2016). After this process, chitin breaks down into ammonia, which can be assimilated by plants.

In agriculture, chitin and its deacetylated derivative chitosan were applied as soil amendment or seed/foliar spray, to improve crop productivity and protection against pathogens (Malerba and Cerana, 2019). Previous studies have suggested that chitin soil amendment could increase plant growth promoting rhizobacteria (PGPR) and fungi (PGPF), and that the stimulation of PGPR and PGPF could act antagonistically against plant pathogens or directly promote plant growth (De Tender et al., 2019). Chitin and its fragments (i.e., chitin oligosaccharides) are also general elicitors known as microbe- or pathogen-associated molecular patterns (MAMPs or PAMPs, respectively) that can be recognized by plant cell-surface localized pattern recognition receptors (PRRs), and subsequently induce pattern-triggered immunity (PTI) (Iriti and Faoro, 2009; Newman et al., 2013). Such defense responses include the production of reactive oxygen species (ROS), as well as biosynthesis of pathogenesis-related (PR) proteins and other antimicrobial compounds (Newman et al., 2013). In *Arabidopsis thaliana*, the cell surface receptor AtLYK5 is the primary receptor for chitin, which forms a chitin-induced complex with AtCERK1 to induce plant immunity (Cao et al., 2014). In this study, we further investigate chitin’s effect as soil amendment, focusing on the growth promotion through monitoring plant physiology and gene expression using both RNA-sequencing (RNA-Seq) and metabolomics analysis. Lettuce growth was monitored every two weeks during its whole growth period of eight weeks. Leaf number, chlorophyll content, flavonoids content and crop weight were measured. RNA-Seq of roots sampled at 72 hours post‐transplanting (hpt) into chitin-amended soil was performed to reveal the early reaction of lettuce roots to chitin treatment. Additionally, metabolomic analyses were done to investigate changes on a metabolomic level after seven days.

## 2 Materials and methods

### 2.1 Soil and lettuce seedling preparation

Chitin flakes obtained from crab shell were purchased from BioLog Heppe GmbH (lot: 40201609; Landsberg, Germany). Peat-based potting soil (Saniflor, Beroepspotgrond, NPK 12-14-24) was purchased from local gardening stores (AVEVE Lammens, Wetteren, Belgium). Potting soil without chitin addition was used directly as control (PS). Chitin-amended soil was potting soil mixed with 2 g L^-1^ chitin (PS+CH). Both treatments were wetted with ground water to reach 40% water filled pore space (WFPS) and incubated in a closed bag in the greenhouse for one week before using.

Lettuce seedlings were germinated from pelletized butterhead lettuce seeds (*L. sativa* L. var. capitata ‘Alexandria’) obtained from Rijk Zwaan Distribution B.V. (De Lier, the Netherlands). First, peat-based sowing soil (Saniflor, Potground voor zaaien en stekken) was used to fill 77-well germination trays, then, one pelleted seed was gently pressed down with tweezers in the center of each well and covered with another thin layer of sowing soil. Every two days the soil was watered to 40% WFPS. Seedlings were transplanted at three to four true leaf stage in 1.3 L pots filled with 1 L potting soil, with or without chitin addition, and grown in the greenhouse at ILVO. Temperature, humidity, photoperiod and light intensity were not strictly controlled and fluctuated along the local weather (Belgium, January – March 2021).

### 2.2 Lettuce physiology measurement

Seedlings of three to four true leaves stage (3-week-old) were transplanted into 1.3 L pots filled with 1 L potting soil. The soil moisture was adjusted to 40% WFPS every week. Chlorophyll and flavonoids content was measured using the Dualex leaf clip sensor (Goulas et al., 2004). Number of leaves was counted every two weeks post-transplanting (wpt). Lettuce heads were harvested and weighed at 8 wpt. Dry weight was measured after drying the fresh head at 60°C for three days.

### 2.3 RNA extraction and gene expression analysis

Lettuce roots and leaves for both treatments (PS, PS+CH) were sampled at 72 hpt. Per treatment five replicates were measured. Each replicate was a pool consisting of five randomly selected plants. In total 25 plants were measured per treatment. Roots and leaves were washed to remove soil, dried on tissue, then flash frozen in liquid nitrogen and stored at -80°C before further use. Total RNA was extracted using the CTAB method as described in detail by Luypaert et al. (2017). Contaminating DNA was removed using DNA-*free* DNA removal kit (AM1906, Invitrogen; Thermo Fisher Scientific, Inc., Waltham, USA, Massachusetts) according to the manufacturer’s protocol.

To study the overall transcriptional reprogramming in lettuce upon chitin treatment, in total five RNA samples per treatment sampled at 72 hpt were shipped to BGI Tech Solutions Co. Ltd. (Hong Kong, China) for cDNA synthesis, library preparation, and sequencing. Strand specific mRNA sequencing was performed on a DNBSEQ platform (MGI Tech Co., Ltd, Shenzen, China). Sequence reads quality of the raw files obtained from BGI was assessed using FastQC v0.11.9 (Andrew, 2015). Reads were then aligned with STAR v2.7.10 (Dobin et al., 2013) against the genome of *L. sativa* cv. ‘Salinas’. The genome version for the *fasta* and the *gtf* file was v7 downloaded from https://www.ncbi.nlm.nih.gov/assembly/GCF_002870075.2. The alignment quality was checked with Qualimap v2.2.1 (Okonechnikov et al., 2015). All steps were carried out on the high-performance computer (HPC) Earth cluster of the School for Life Sciences and Facility Management at ZHAW.

Read counting was performed using featureCounts v2.0.1 (Liao et al., 2014). To analyze the different gene expression the featureCounts output was analyzed with R v4.2.1 (Team, 2020) using the DESeq2 package v1.30.1 (Love et al, 2014) in RStudio v1.3.959 (RStudio Team, 2019). As a log_2_-fold change threshold one was used, the adjusted *p*-value was set to 0.05. A statistical overrepresentation test was performed using PANTHER v17.0 (www.pantherdb.org; Mi et al., 2019) by applying Fisher’s exact test and correction for false discovery rate (FDR). Filtering and visualizing of the output were performed according to Bonnot et al., (2019). An enrichment analysis of the Kyoto Encyclopedia of Genes and Genomes (KEGG, release 103) pathways was conducted using clusterProfiler v4.4.4 (Wu et al., 2021). The cut off was set to a *p*-value of 0.05. Pathway visualization was performed using R v4.2.1 (Team, 2020) and RStudio 1.3.959 (RStudio Team, 2019). First an organism package was created using AnnotationForge v1.36.0 (Carlson and Pagès 2023). The mapping against the KEGG pathways was performed with the pathview R package v1.36.0 (Luo and Brouwer, 2013).

In addition, the expression of several known defense related genes in lettuce and DEGs selected from RNA-Seq analysis were studied by RT-qPCR (described in detail in **Supplementary Text S1**), using the method described previously (De Keyser et al., 2020).

### 2.4 Phenolic compounds analysis

For determination of PCs, seedlings were transplanted in 0.9 L pots. Roots and leaves were sampled at 1 wpt. To collect enough material, 25 plants from the same treatment were randomly selected and pooled as one biological replicate. In total four bio-replicates per treatment were assayed. Leaf and root tissues were grinded immediately after sampling to a fine powder with liquid nitrogen using mortar and pestle, then freeze-dried and sealed in a vacuumed bag until further use. PCs were extracted following an in-house developed two-step extraction protocol using methanol (De Paepe et al., 2014; Kips et al., 2017). For each sample, 500 mg of freeze-dried tissue powder was weighed and transferred to a 50-mL tube and 50 µL internal standard (daidzin, 100ng µL^-1^) was added. To extract the PCs, 10 mL pure methanol was added, vortexed for 1 min, then sonicated using Elma Transsonic digitial S (Elma Schmidbauer GmbH, Germany) at 40 kHz for 15 min. The suspension was centrifuged at 3,000 *g* for 5 min, the obtained supernatant was transferred to a new glass tube and stored at 4°C. The second extraction was done following the same procedure using 20% (*v*/*v*) methanol. For samples that weighed less than 500 mg, the volume of internal standard (or extraction solvents) was adapted according to the sample weight to internal standard (or solvent) volume ratio. The two-step extractions were collected in the same tube for each sample and filtered with 0.22 µm PVDF syringe filter. Samples were analyzed with both, targeted approach using liquid chromatography with tandem mass spectrometry (LC-MS/MS) and untargeted approach using liquid chromatography-high resolution mass spectrometry (LC-HRMS).

For LC-MS/MS analysis 5 µL of the final extract was injected onto an Acquity UPLC BEH Shield RP18 column (2.1 × 150 mm; 1.7 µm) and analyzed using an Acquity Ultra Performance liquid chromatograph (Waters, Milford, MA, USA) coupled to a Xevo TQ-XS mass spectrometer (Waters) operated in negative electrospray. Details of the used LC mass spectrometric method are described by Kips et al. (2017). Quantification was done using external calibration curves. Data recording was done in MRM-mode by MassLynx v4.1 while the integration was performed with TargetLynx v4.1 (Waters).

The same extracts were also analyzed by LC-HRMS (Synapt G2-S, Waters) with an untargeted approach in both positive and negative electrospray. Data recording was done in a data independent mode (MSe-mode) using Masslynx. For quality control purposes a mixture of equally amounts of all obtained extracts of either leaves or roots (QC) were made and analyzed throughout the run. All samples were randomized prior to LC-HRMS analysis. For data processing Progenesis Qi v2.4.6911.27652 (Waters) was used to perform peak picking, sample alignment, deconvolution, and principal component analysis (PCA) to assess the interrelations between chitin-treated and untreated samples.

## 3 Results

### 3.1 Measurements of physiological parameters

Two and four weeks after transplanting, no significant difference in number of leaves or chlorophyll content was noticed for the chitin-treated lettuce plants, except that at 2 wpt the flavonoids content was increased compared to the control plants. At 6 and 8 wpt, chitin-treated plants showed a significant increase in growth, with more leaves and higher chlorophyll content. By the end of the eight-week growth period, the chitin-treated lettuce plants had 89.5% more fresh weight and 61.2% more dry weight compared to the control plants (*p*< 0.05). For the chitin-treated lettuce, it appeared that past the heading stage the growth was promoted whereas the flavonoids content was decreased (**Table 1**).

**Table 1:** Overview of the physiological parameters measured every two weeks post‐transplanting (wpt) for lettuce plants in chitin-amended potting soil (PS+CH) and in non-treated potting soil as control (PS).

Measurement	PS	PS+CH	Weeks post-transplanting	*p*-value
Flavonoid content^*^	0.14 ± 0.01	0.15 ± 0.01	2	**0.02**
	0.25 ± 0.02	0.23 ± 0.01	4	0.33
	0.52 ± 0.07	0.38 ± 0.04	6	**< 0.01**
	0.81 ± 0.14	0.58 ± 0.08	8	**0.01**
Chlorophyll content (µg·cm^-2^)	11.01 ± 0.45	10.85 ± 0.68	2	0.66
	15.49 ± 0.33	15.75 ± 0.72	4	0.50
	16.77 ± 1.34	19.68 ± 0.50	6	**< 0.01**
	10.20 ± 2.65	14.65 ± 0.49	8	**0.03**
Leaf number	10.00 ± 0.58	9.83 ± 1.07	2	0.77
	24.50 ± 1.26	25.83 ± 2.11	4	0.26
	39.00 ± 3.42	47.00 ± 1.91	6	**< 0.01**
	51.80 ± 2.64	60.00 ± 3.52	8	**0.01**
Fresh weight (g)	74.85 ± 7.81	141.86 ± 6.12	8	**< 0.01**
Dry weight (g)	9.37 ± 0.95	15.10 ± 3.42	8	**0.03**

*: Flavonoids content was given in relative absorbance units, by analyzing the screening effect of flavonoids on chlorophyll fluorescence, thus has no unit.

The different measured parameters are in the first column. Columns two and three show the different treatment groups. Column four indicates the time of measurement. The last column shows the *p*-value of the *t*-test between the two treatment groups for the corresponding measurement. Significant differences (*p* < 0.05) are marked in bold. For each measurement, *n* = 6.

### 3.2 Gene ontology enrichment

**Figure 1:**
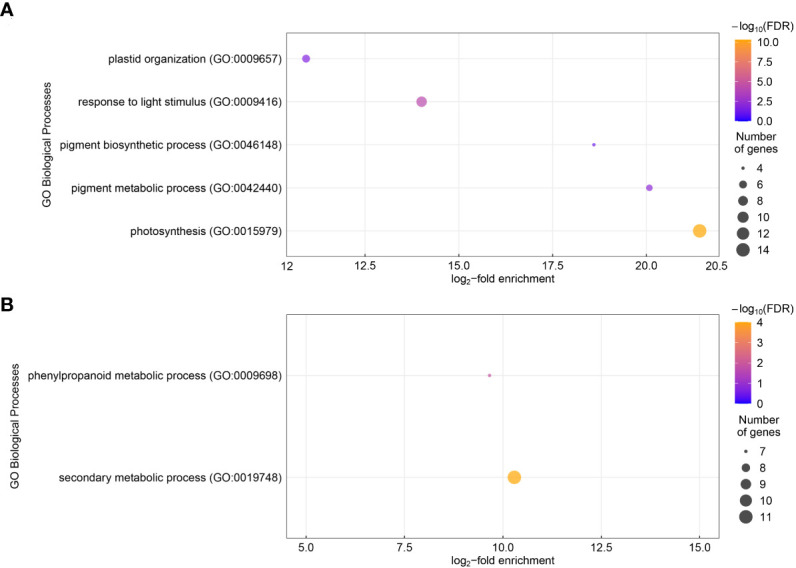
Gene Ontology (GO) biological processes enrichment of the differentially expressed genes (DEGs) for lettuce plants grown in chitin-amended soil in comparison to non-treated control plants. The log_2_-fold enrichment of GO biological processes are displayed for the upregulated genes **(A)** and the downregulated genes **(B)** in chitin-treated lettuce plants. The color of the dots shows the -log_10_ of the false detection rate (FDR). The dot size is proportional to the gene number for each GO category.

In total, 321 genes showed significantly different expression levels in roots from the chitin-treated plants compared to the control plants (log_2_-fold change ≥ 1 or ≤ -1, FDR< 0.05) (**Supplementary Table S1**). Of these differentially expressed genes (DEGs), 214 were lower expressed upon chitin treatment and 107 had a higher expression level. However, a number of these genes had rather low read counts, therefore they were not verified using RT-qPCR (**Supplementary Text S1**). A Gene Ontology (GO) overrepresentation test, for biological processes, of the higher expressed genes showed an enrichment of five GO terms, namely plastid organization (GO: 0009657), response to light stimulus (GO: 0009416), pigment biosynthetic process (GO: 0046148), pigment metabolic process (GO: 0042440) and photosynthesis (GO: 0015979) (**Figure 1A**). The strongest enrichment for the higher expressed genes was for the photosynthesis which contained 14 genes and showed a log_2_-fold enrichment of 21.4. The GOs for pigment metabolic processes and pigment biosynthetic process were the second and third strongest enriched terms with a log_2_-fold change of 20.1 and 18.6, respectively. Both however contained less genes (five and four genes, correspondingly) than the photosynthesis category. Response to light stimulus and plastid organization showed a log_2_-fold enrichment of 14.0 and 10.9, respectively. Lower expressed genes were enriched in secondary metabolic processes (GO: 0019748) and phenylpropanoid metabolic process (GO:0009698), with log_2_-fold enrichment of 10.3 and 9.7 (**Figure 1B**). In lettuce leaves, 15 genes showed significant different expression levels (**Supplementary Table S2**). The GO enrichment did not show any biological process that was over-represented.

### 3.3 KEGG pathway enrichment

The KEGG enrichment for the stronger expressed genes in the roots of the chitin-treated plants showed four pathways which were enriched (**Table 2**). The photosynthesis pathway lsv00195 was the one with the highest significance. Related to the photosynthesis, the pathway for the photosynthesis antenna proteins lsv00196 was also enriched. The porphyrin metabolism lsv00860 also showed a significant enrichment for the chitin-treated plants. The only pathway which was enriched independent of the photosynthesis was the flavonoid pathway lsv00941. In total seven pathways were enriched in the downregulated gene set (**Table 2**). The only pathway that was also present in the GO and KEGG enrichment for the downregulated genes was the phenylpropanoid biosynthesis lsv00940. For lettuce leaves, no KEGG pathway was enriched.

**Table 2:** Enriched KEGG pathways for the up- and downregulated gene sets observed for the lettuce plants in chitin-amended potting soil (PS+CH) in comparison to non-treated control plants (PS).

Gene set	KEGG pathway	Description	Adjusted *p*-value
Upregulated	lsv00195	Photosynthesis	< 0.01
	lsv00941	Flavonoid biosynthesis	< 0.01
	lsv00860	Porphyrin metabolism	< 0.01
	lsv00196	Photosynthesis - antenna proteins	0.04
Downregulated	lsv00073	Cutin, suberine and wax biosynthesis	< 0.01
	lsv00950	Isoquinoline alkaloid biosynthesis	< 0.01
	lsv02010	ABC transporters	< 0.01
	lsv00071	Fatty acid degradation	< 0.01
	lsv04146	Peroxisome	< 0.01
	lsv00940	Phenylpropanoid biosynthesis	< 0.01
	lsv00350	Tyrosine metabolism	< 0.01

The first column on the left contains the two different gene sets. The second and third column show the pathway abbreviation from KEGG and the corresponding pathway name. The last column displays the adjusted *p*-value of the KEGG enrichment.

### 3.4 Pathway visualization

Since the phenylpropanoid pathway was present in the GO and KEGG enrichment, this pathway was further investigated and visualized. Next to the phenylpropanoid pathway also the downstream flavonoid pathway and the photosynthesis pathway were considered in the RNA-Seq analysis of roots. Significant DEGs were mapped against the pathways and visualized. The visualization of the phenylpropanoid biosynthesis pathway shows that all mapped genes that were significantly downregulated mapped to the KEGG ontology 1.11.17 (**Supplementary Figure S1**). Genes mapped to this KEGG ontology were all downregulated peroxidases, connecting the phenylpropanoid pathway to the lignin pathway (**Table 3**). The mapped genes act further downstream the phenylpropanoid pathway connecting it to the lignin pathway. The downstream flavonoid pathway analysis reports a different situation (**Supplementary Figure S2**). In contrast to the upstream phenylpropanoid pathway, which showed a lower expression than the control group, the flavonoid biosynthesis shows indeed a higher expression than the non-chitin-treated group. A total of five DEGs mapped to the flavonoid biosynthesis pathway, and all were upregulated (**Table 3**). The mapped genes included two that encode different chalcone synthases (NCBI Gene ID 111882072 and NCBI Gene ID 111883451, log_2_-fold change 6.86 and 4.69, respectively), which were mapped to the KEGG ontology 2.3.1.74 and mark the connection between the phenylpropanoid biosynthesis and the flavonoid biosynthesis. The gene encoding the chalcone synthase 11882072 showed the highest log_2_-fold change of all mapped genes. The gene with the second highest log_2_-fold change (5.019) encodes a dihydroflavonol 4- reductase (NCBI gene ID 111897350) that is needed to produce fustin. Further DEG encoded a chalcone isomerase-like protein 2 (NCBI Gene ID 111919658) and a flavonoid 3’-monooxygenase (NCBI Gene ID 111891078). In total seven of the higher expressed genes in the chitin-treated group were mapped to the KEGG ontology of the lsv00195 pathway for photosynthesis (**Supplementary Figure S3**). Three genes encode proteins belonging to the photosystem I while three genes encode proteins belonging to the photosystem II. The last gene was a ferredoxin-NADP^+^ reductase-encoding gene (NCBI Gene ID 111897632) (**Table 3**).

**Table 3:** Overview of three KEGG pathways and the mapped differently expressed genes (DEG) in chitin-treated lettuce plants (PS+CH) in comparison to untreated control plants (PS).

Pathway	NCBI gene ID	Description	log_2_-fold change
Phenylpropanoid	111877700	Peroxidase 51	-1.18
	111878787	Peroxidase 4	-1.02
	111891404	Peroxidase 12	-1.44
	111894964	Peroxidase 11	-1.63
	111910447	Peroxidase 11	-1.72
	111919669	Peroxidase P7	-1.39
Flavonoid	111882072	Chalcone synthase J	6.86
	111883451	Chalcone synthase	4.69
	111891078	Flavonoid 3’-monooxygenase	1.52
	111897350	Dihydroflavonol 4-reductase	5.02
	111919658	Chalcone isomerase-like protein 2	1.74
Photosynthesis	111881229	Photosystem I reaction center subunit IV A, chloroplastic	1.04
	111897400	Photosystem II 22 kDa protein, chloroplastic	1.13
	111897632	Ferredoxin–NADP reductase, leaf-type isozyme, chloroplastic	1.44
	111909181	Photosystem I reaction center subunit VI, chloroplastic	1.07
	111912546	Photosystem II reaction center PSB28 protein, chloroplastic	1.59
	111915097	Photosystem II reaction center W protein, chloroplastic	1.18
	111918291	Photosystem I reaction center subunit XI, chloroplastic	1.12

The first column represents the different pathways. In the second and third column the NCBI gene ID and the description of the genes are shown. The last column displays the log_2_-fold change of the gene expression of PS+CH compared to PS.

### 3.5 Phenolic compounds analysis

The targeted approach using LC-MS/MS detected 19 and 16 PCs in lettuce leaves and roots, respectively (**Table 4**). In roots, for example, the amount of salicylic acid and chlorogenic acid was significantly higher for the chitin-treated plants than roots of the control plants (**Table 4**). In the leaves, the concentration of salicylic acid (SA), *p*-coumaric acid (*p*-CA) and ferulic acid (FA) was higher for the chitin-treated plants, while the concentration of cynaroside was higher in control plants (**Table 4**).

**Table 4:** Phenolic compounds (mg kg^-1^ ± sd %) determined using LC-MS for chitin-treated lettuce plants (PS+CH) and for untreated control plants (PS).

Phenolic compounds		Leaf		Root	
		PS	PS+CH	PS	CH
Phenolic acid	Salicylic acid*	0.22 ± 2.51%	0.35 ± 16.92%	1.24 ± 26.69%	2.77 ± 26.29%
	4-OH-phenylacetic acid	–	–	26.74 ± 19.77%	28.05 ± 16.67%
	Protocatechuic acid	0.32 ± 8.41%	0.31 ± 5.23%	6.91 ± 21.87%	6.15 ± 32.24%
	*p*-Coumaric acid^#^	0.11 ± 8.11%	0.16 ± 10.64%	0.50 ± 21.26%	0.51 ± 18.51%
	Caffeic acid	19.15 ± 9.03%	26.67 ± 19.30%	26.08 ± 27.99%	34.20 ± 12.16%
	Quinic acid	143.82 ± 8.59%	125.85 ± 8.19%	923.25 ± 37.37%	1,081.47 ± 21.24%
	Ferulic acid^#^	0.09 ± 7.34%	0.15 ± 17.44%	0.75 ± 17.66%	0.73 ± 14.13%
	Clorogenic acid^&^	1,509.19 ± 11.52%	1,400.58 ± 4.29%	3,047.58 ± 14.97%	4,115.31 ± 10.48%
	Chicoric acid	24,921.23 ± 27.37%	24,882.23 ± 5.02%	65,879.13 ± 16.80%	73,768.17 ± 15.84%
Flavonoids	Apigenin	0.01 ± 4.04%	–	0.005 ± 92.91%	0.01 ± 67.15%
	Naringenin	0.004 ± 19.69%	0.006 ± 22.24%	0.007 ± 88.18%	0.06 ± 83.58%
	Luteolin	0.11 ± 13.64%	0.10 ± 20.28%	0.17 ± 16.73%	0.17 ± 31.23%
	Quercetin	0.20 ± 14.14%	0.21 ± 31.80%	–	–
	Isorhamnetin	0.001 ± 22.11%	0.001 ± 35.52%	–	–
	Apigetrin	0.04 ± 15.52%	0.03 ± 15.71%	–	–
	Avicularin	0.02 ± 14.08%	0.01 ± 3.00%	0.04 ± 1.56%	–
	Phloridzin	0.05 ± 13.09%	0.04 ± 11.50%	0.36 ± 48.73%	0.39 ± 27.93%
	Cynaroside^#^	4.80 ± 11.89%	3.75 ± 11.26%	0.92 ± 28.21%	1.18 ± 45.01%
	Isoquercetin	6.97 ± 18.54%	6.00 ± 24.22%	26.10 ± 18.89%	28.72 ± 39.35%
	Quercetin-3-O-glucuronide	153.92 ± 5.60%	169.09 ± 10.53%	872.57 ± 31.43%	1,037.18 ± 27.68%
	Rutin	6.40 ± 19.07%	4.95 ± 7.39%	18.41 ± 30.58%	20.98 ± 35.67%

*: compounds significantly different in both and leaf. “-”: not detected. ^#^: compounds significantly different in leaf. ^&^: compounds significantly different in root.

Untargeted analysis using LC-HRMS in both positive ionization (ESIpos) and negative ionization (ESIneg) each detected 5,833 and 9,308 features in all samples. Profiles of both ESIpos and ESIneg showed clear difference between root and leaf samples. To further investigate the differences between treated and non-treated plants, root and leaf samples were processed separate from each other, and only those featured ions that showed a variation coefficient below 30% in their respective QC samples were kept for further analysis. Next a supervised pattern recognition technique (OPLS-DA) was used to maximize the differences between the chitin-treated and non-treated plants. Features which showed both the biggest and the most significant difference between the two treatments were selected for PCA plot construction. In total, 22 and 104 features in leaf and root, respectively, showed a clear difference upon chitin treatment in ESIpos whereas ESIneg showed 90 and 69 features, respectively (**Supplementary Figure S4**).

### 3.6 RT-qPCR

Among 38 target genes, 14 showed expression in all root samples, the rest were not analyzed due to the high quantification cycle (*C_q_*) values (>35), indicating low starting concentration of the target nucleic acids. Although not significant, most of these genes showed slightly higher expression in roots of chitin-treated plants in 72 hpt samples (**Supplementary Text S1**). These genes were found differentially expressed in RNA-Seq with log_2_Fold change< 1.

## 4 Discussion

Chitin as soil amendment promoting plant growth has been reported before (Muymas et al., 2015; Debode et al., 2016; Shamshina et al., 2020; De Tender et al., 2021). Our results showed that lettuce grown in crab chitin-amended soil gained almost 90% increase in fresh weight compared to the control, which is higher than reported previously (Debode et al., 2016). Also, the chlorophyll content and the number of leaves were significantly increased for the chitin-amended plants from 6 wpt onwards. Temperature, relative humidity and irradiance are all important factors affecting plant growth (Tibbitts and Bottenberg, 1976; Mortensen, 1986; Ahmed et al., 2020). In this study, instead of using a growth chamber with strictly controlled environmental conditions, we went one step further. We evaluated the effect of chitin soil treatment in the greenhouse, where environmental factors can fluctuate much like in commercial lettuce production greenhouses. Although the growth conditions were less controlled compared to previous studies, chitin’s growth promoting effect on lettuce was still significantly contrasted to non-treated plants. Thus, the growth promoting effect of chitin appears not to be dependent on strict environmental conditions of temperature, light or humidity.

De Tender et al. (2019) demonstrated that the addition of chitin in potting soil can lead to a higher availability of nitrogen i.e., $NO_{3}^{-}$ and $NH_{4}^{+}$ . This in turn can cause an activation of ammonium-oxidizers that will convert ammonium to $NO_{2}^{-}$ and $NO_{3}^{-}$ providing more nutrients to the plant. Chitin amendment also increases the abundance of PGPR and PGPF in the lettuce rhizosphere (Hallmann et al., 1999; Debode et al., 2016). Plants release a substantial fraction of their photo-assimilated carbon through their roots (Kaiser et al., 2015). Since chitin promotes chitin catabolic organisms, ammonium-oxidizers, PGPR and PGPF this could lead to a higher demand of photo-assimilated carbon and therefore to a higher cellular respiration in the roots. Aschan and Pfanz (2003) showed that roots can recycle CO_2_ released from cellular respiration, which might explain the increased transcription of photosynthesis related genes in lettuce roots in this study.

Three days after transplanting, lettuce grown in chitin-amended soil showed an upregulation of flavonoids biosynthesis genes in their roots. Genes that play an important role in flavonoids biosynthesis, such as different chalcone synthases showed a higher expression in chitin-treated plants compared to non-treated plants. Additionally, MYB111, a transcription factor found to regulate flavonoid biosynthesis in *A. thaliana*, was also higher expressed in chitin-treated plants compared to non-treated plants (log_2_-fold change >5, *p*_adj_ = 0.00003) (Li et al., 2019). Similar findings were already made by Akiyama et al. (1994) who demonstrated that partially *N*-deacetylated chitin strongly induced antimicrobial flavonoid production in pea epicotyls. Further, it was shown in *A. thaliana* that the expression of MYB domain-containing transcription was induced upon chitin treatment (Libault et al., 2007). However, the early upregulation in the roots of these flavonoid biosynthesis related genes was not found in the leaves of chitin-treated lettuce. The increased flavonoid levels in the leaves at a later stage (2 wpt) suggests that there might be a lag in the upregulation of flavonoid biosynthesis in the leaves due to long‐distance signaling (Takahashi and Shinozaki, 2019), or that this pathway upregulation could be only root localized by the direct contact with chitin. In the latter scenario, flavonoids accumulation would first happen in roots, and then move over long distances to leaves (Buer et al., 2008). We tried to verify the expression levels of *MYB111* and the chalcone synthase gene by RT-qPCR. Both genes had a low base mean in roots and consequently high *C_q_* values (>35). The expression of these genes in lettuce might be very low and sensitive to timing. The changes in expression over time can also be observed by the flavonoid levels in the leaves. Only at 2 wpt, the flavonoid levels in lettuce leaves were significantly higher in chitin-treated plants compared to non-treated plants. Already at 4 wpt, the flavonoid content in leaves of lettuce grown in chitin-amended soil was lower compared to the control group. The synthesis of flavonoids and lignin are two downstream bifurcations of the phenylpropanoid pathway. Kandel et al. (2022) observed that the lignin pathway is upregulated in lettuce roots 24 h after chitin application to the growing media. Especially the peroxidases connecting the phenylpropanoid pathway to the lignin pathway showed an increase in transcript levels. Our analysis showed that these peroxidases were downregulated 72 hpt in lettuce roots. It is known that in *A. thaliana* and *Medicago sativa* the downregulation of the lignin pathway leads to a change of flux towards the flavonoid pathway (Besseau et al., 2007; Gallego-Giraldo et al., 2011). These varying results suggest that chitin affects the regulation of the peroxidases at the end of the phenylpropanoid pathway, in a time sensitive manner. Upregulation or downregulation of these peroxidases leads to a change of flux towards the lignin or the flavonoid pathway, respectively.

To test if the detected transcriptional changes in lettuce correlate with changes in the compounds produced by the encoded enzymes, a metabolomics approach was followed. Since the upregulation of the flavonoid biosynthesis pathway was found three days after transplanting of seedlings, without knowing how fast lettuce accumulates relative metabolites, it was decided to use plants at 1 wpt. With the targeted approach using LC-MS, 21 out of 46 (**Supplementary Table S3**) reference compounds were found present in lettuce plants. PCs that showed significant difference in chitin-treated plants (SA, *p*-CA, chlorogenic acid and FA) are all phenolic acids, while all flavonoids detected showed relatively low concentrations and no significant difference with the control plants (PS). The detected flavonone that showed a significant difference between treated and non-treated plants was cymaroside, which was higher concentrated in the roots of untreated plants. Chlorogenic acid and *p*-CA were more abundantly detected in the roots of chitin-treated plants. Both compounds have been reported to have antifungal properties and are induced as defense response against fungal plant pathogens (Wojciechowska et al., 2014; Martínez et al., 2017; Islam et al., 2019; Yuan et al., 2019; Liu et al., 2020; Zhang et al., 2020). Since the fungal cell wall is mostly made up of chitin (Bowman and Free, 2006), it makes sense that chitin flakes would trigger the same response upon detection by the plant. The only targeted compound of the LC-MS analysis that showed a significant increase upon chitin treatment in both roots and leaves was SA. SA is a hormone that is essential for plant defense (Ding and Ding, 2020). It promotes local immune responses and plays an important role in the basal defense against (hemi-)biotrophic pathogens. It is also important for the establishment of systemic acquired resistance (Peng et al., 2021). SA is needed for PTI as well as for the effector-triggered immunity (Li et al., 2019). Although we were not able to detect an upregulation of plant defense related genes, our results suggest that chitin soil amendment activates PTI in lettuce. Defense-related genes most likely were not detected due to the late sampling point (after three days). We were, however, able to detect higher levels of defense-associated metabolites in both leaf and root tissues, such as SA. Furthermore, SA is known to increase flavonoid production in lettuce (Moreno-Escamilla et al., 2020). This finding is congruent with our results of the flavonoid pathway being upregulated in the roots, higher flavonoid levels in the leaves within the first two weeks and higher levels of SA upon chitin treatment. At four weeks, the levels of flavonoids decreased in chitin-treated plants. This decrease may also indicate an earlier drop in SA levels. This short activation of plant defense upon chitin treatment indicates a defense priming effect, which is a low-cost defensive measure where plant defense responses are only slightly activated upon treatment but would enable plants to mount a faster and/or stronger defense response upon subsequent challenge (Mauch-Mani et al., 2017). For example, the overall gene expression in eight-weeks-old strawberry was not changed upon mere chitin amendment, however, when challenged with a fungal pathogen, a significant upregulated gene expression was observed in chitin-treated strawberry plants (De Tender et al., 2021). In addition, leaves from chitin-treated lettuce also showed a much higher apoplast ROS burst than the control upon elicitor treatment, indicating potential priming effect (our unpublished data). Chitin as soil amendment hence caused a priming effect in lettuce seedlings, which resulted in the upregulation of flavonoid biosynthesis, accumulation of PCs. After the initial priming state, due to the defense-growth trade-off, when chitin-treated plants were promoted for growth (six weeks), it is thus reasonable to observe reduced defense related flavonoids content (He et al., 2022). This priming effect possibly have long‐lasting defense effect and can be further verified by gene expression assay upon pathogen challenge in later growth stage. Chitin remains a promising organic substrate to promote plant growth and defenses. Whether this activation is due to chitin perception by the plant or due to a possible (direct or indirect) change in the lettuce rhizosphere microbiome upon treatment remains to be investigated.

## Data availability statement

The data presented in the study are deposited in the NCBI GEO repository, accession number GSE224725.

## Author contributions

LL, MM, MK, CZ, BC, and JP conceptualized the study. LL, MK, EDK, and CVP created the methodology and curated the data. LL, MK, EDK, and CVP analyzed the data. LL, MK, BC, and JP were involved in the investigations. LL, MK, and CVP contributed to the data visualization. MU, CZ, BC, and JP were involved in funding acquisition. LL, MK, CVP, EDK, BC, and JP prepared the original draft with assistance from MM, MU, and CZ for review and editing. All authors contributed to the article and approved the submitted version.

## References

[B1] Ahmed H. A. Yu-Xin T. Qi-Chang Y. (2020). Optimal control of environmental conditions affecting lettuce plant growth in a controlled environment with artificial lighting: A review. S. Afr. J. Bot. 130, 75–89. doi: 10.1016/j.sajb.2019.12.018

[B2] Akiyama K. Kawazu K. Kobayashi A. (1994). Partially N-deacetylated chitin elicitor induces antimicrobial flavonoids in pea epicotyls. Z. Naturforsch. C 49, 811–818. doi: 10.1515/znc-1994-11-1217

[B3] Andrew S. (2015). FastQC: a quality control tool for high throughput sequence data (Babraham Bioinformatics). Available online at: https://www.bioinformatics.babraham.ac.uk/projects/fastqc/.

[B4] Andronopoulou E. Vorgias C. E. (2004). Multiple components and induction mechanism of the chitinolytic system of the hyperthermophilic archaeon *Thermococcus chitonophagus. Appl* . Microbiol. Biotechnol. 65, 694–702. doi: 10.1007/s00253-004-1640-4 15322771

[B5] Aschan G. Pfanz H. (2003). Non-foliar photosynthesis – a strategy of additional carbon acquisition. Flora: Morphol. 198, 81–97. doi: 10.1078/0367-2530-00080

[B6] Besseau S. Hoffmann L. Geoffroy P. Lapierre C. Pollet B. Legrand M. (2007). Flavonoid accumulation in *Arabidopsis* repressed in lignin synthesis affects auxin transport and plant growth. Plant Cell 19, 148–162. doi: 10.1105/tpc.106.044495 17237352 PMC1820963

[B7] Bonnot T. Gillard M. Nagel D. (2019). A simple protocol for informative visualization of enriched gene ontology terms. BioProtoc 9, 3429. doi: 10.21769/BioProtoc.3429

[B8] Bowman S. M. Free S. J. (2006). The structure and synthesis of the fungal cell wall. Bioessays 28, 799–808. doi: 10.1002/bies.20441 16927300

[B9] Buer C. S. Muday G. K. Djordjevic M. A. (2008). Implications of long-distance flavonoid movement in *Arabidopsis thaliana* . Plant Signal Behav. 3, 415–417. doi: 10.4161/psb.3.6.5440 PMC2634320 19704584

[B10] Carlson M. Pagès H.. (2023). AnnotationForge: Tools for building SQLite-based annotation data packages. R package version 1.40.1. Available online at: https://bioconductor.org/packages/AnnotationForge

[B11] Cao Y. Liang Y. Tanaka K. Nguyen C. T. Jedrzejczak R. P. Joachimiak A.. (2014). The kinase LYK5 is a major chitin receptor in *Arabidopsis* and forms a chitin-induced complex with related kinase CERK1. Elife 3, e03766. doi: 10.7554/eLife.03766 PMC4356144 25340959

[B12] Chen J. Lü S. Zhang Z. Zhao X. Li X. Ning P.. (2018). Environmentally friendly fertilizers: a review of materials used and their effects on the environment. Sci. Total. Environ. 613-614, 829–839. doi: 10.1016/j.scitotenv.2017.09.186 28942316

[B13] Dahiya N. Tewari R. Hoondal G. S. (2006). Biotechnological aspects of chitinolytic enzymes: a review. Appl. Microbiol. Biotechnol. 71, 773–782. doi: 10.1007/s00253-005-0183-7 16249876

[B14] Debode J. De Tender C. Soltaninejad S. van Malderghem C. Haegeman A. van der Linden I.. (2016). Chitin mixed in potting soil alters lettuce growth, the survival of zoonotic bacteria on the leaves and associated rhizosphere microbiology. Front. Microbiol. 7, 565. doi: 10.3389/fmicb.2016.00565 27148242 PMC4838818

[B15] De Keyser E. Desmet L. Losschaert M. De Riek J. (2020). “A general protocol for accurate gene expression analysis in plants,” in Quantitative real-time PCR. Eds. Biassoni R. Raso A. (New York, NY: Humana), 105–118. 10.1007/978-1-4939-9833-3_9 31578691

[B16] De Paepe D. Valkenborg D. Noten B. Servaes K. Diels L. de Loose M.. (2014). Variability of the phenolic profiles in the fruits from old, recent and new apple cultivars cultivated in Belgium. Metabolomics 11, 739–752. doi: 10.1007/s11306-014-0730-2

[B17] De Tender C. Mesuere B. van der Jeugt F. Haegeman A. Ruttink T. Vandecasteele B.. (2019). Peat substrate amended with chitin modulates the N-cycle, siderophore and chitinase responses in the lettuce rhizobiome. Sci. Rep. 9, 9890. doi: 10.1038/s41598-019-46106-x 31289280 PMC6617458

[B18] De Tender C. Vandecasteele B. Verstraeten B. Ommeslag S. De Meyer T. De Visscher J.. (2021). Chitin in strawberry cultivation: foliar growth and defense response promotion, but reduced fruit yield and disease resistance by nutrient imbalances. Mol. Plant Microbe Interact. 34, 227–239. doi: 10.1094/MPMI-08-20-0223-R 33135964

[B19] Ding P. Ding Y. (2020). Stories of salicylic acid: A plant defense hormone. Trends Plant Sci. 25, 549–565. doi: 10.1016/j.tplants.2020.01.004 32407695

[B20] Dobin A. Davis C. A. Schlesinger F. Drenkow J. Zaleski C. Jha S.. (2013). STAR: ultrafast universal RNA-seq aligner. Bioinformatics 29, 15–21. doi: 10.1093/bioinformatics/bts635 PMC3530905 23104886

[B21] FAOSTAT (2021) FAOSTAT: food and agriculture data (Food and Agriculture Organization of the United Nations) (Accessed March 02, 2023).

[B22] Gallego-Giraldo L. Jikumaru Y. Kamiya Y. Tang Y. Dixon R. A. (2011). Selective lignin downregulation leads to constitutive defense response expression in alfalfa (*Medicago sativa* l.). New Phytol. 190, 627–639. doi: 10.1111/j.1469-8137.2010.03621.x 21251001

[B23] Goulas Y. Cerovic Z. G. Cartelat A. Moya I. (2004). Dualex: a new instrument for field measurements of epidermal ultraviolet absorbance by chlorophyll fluorescence. Appl. Opt. 43, 4488–4496. doi: 10.1364/ao.43.004488 15382317

[B24] Hallmann J. Rodrıíguez-Kábana R. Kloepper J. W. (1999). Chitin-mediated changes in bacterial communities of the soil, rhizosphere and within roots of cotton in relation to nematode control. Soil Biol. Biochem. 31, 551–560. doi: 10.1016/S0038-0717(98)00146-1

[B25] He Z. Webster S. He S. Y. (2022). Growth-defense trade-offs in plants. Curr. Biol. 32, R634–R639. doi: 10.1016/j.cub.2022.04.070 35728544

[B26] Iriti M. Faoro F. (2009). Chitosan as a MAMP, searching for a PRR. Plant Signal Behav. 4, 66–68. doi: 10.4161/psb.4.1.7408 PMC2634077 19704712

[B27] Islam M. T. Lee B.-R. van La H. Lee H. Jung W.-J. Bae D.-W.. (2019). *p*-coumaric acid induces jasmonic acid-mediated phenolic accumulation and resistance to black rot disease in *Brassica napus* . Physiol. Mol. Plant Pathol. 106, 270–275. doi: 10.1016/j.pmpp.2019.04.001

[B28] Kaiser C. Kilburn M. R. Clode P. L. Fuchslueger L. Koranda M. Cliff J. B.. (2015). Exploring the transfer of recent plant photosynthates to soil microbes: mycorrhizal pathway vs direct root exudation. New Phytol. 205, 1537–1551. doi: 10.1111/nph.13138 PMC4357392 25382456

[B29] Kandel S. L. Anchieta A. G. Shi A. Mou B. Klosterman S.J (2022)Crustacean meal elicits expression of growth and defense-related genes in roots of lettuce and tomato Phyto Front. 2, 10–20 doi: 10.1094/PHYTOFR-03-21-0017-R

[B30] Kips L. De Paepe D. van Meulebroek L. van Poucke C. Larbat R. Bernaert N.. (2017). A novel spiral-filter press for tomato processing: process impact on phenolic compounds, carotenoids and ascorbic acid content. J. Food Eng. 213, 27–37. doi: 10.1016/j.jfoodeng.2017.06.010

[B31] Kumar S. (2012). Biopesticides: A need for food and environmental safety. J. Fertil. Pestic. 3, e107. doi: 10.4172/2155-6202.1000e107

[B32] Li B. Fan R. Guo S. Wang P. Zhu X. Fan Y.. (2019). The *Arabidopsis* MYB transcription factor, MYB111 modulates salt responses by regulating flavonoid biosynthesis. Environ. Exp. Bot. 166, 103807. doi: 10.1016/j.envexpbot.2019.103807

[B33] Li N. Han X. Feng D. Yuan D. Huang L.-J. (2019). Signaling crosstalk between salicylic acid and ethylene/jasmonate in plant defense: Do we understand what they are whispering? Int. J. Mol. Sci. 20, 671. doi: 10.3390/ijms20030671 30720746 PMC6387439

[B34] Liao Y. Smyth G. K. Shi W. (2014). featureCounts: an efficient general purpose program for assigning sequence reads to genomic features. Bioinformatics 30, 923–930. doi: 10.1093/bioinformatics/btt656 24227677

[B35] Libault M. Wan J. Czechowski T. Udvardi M. Stacey G. Wan J.. (2007). Identification of 118 *Arabidopsis* transcription factor and 30 ubiquitin-ligase genes responding to chitin, a plant-defense elicitor. Mol. Plant Microbe Interact. 20, 900–911. doi: 10.1094/MPMI-20-8-0900 17722694

[B36] Liu X. Ji D. Cui X. Zhang Z. Li B. Xu Y.. (2020). *p*-coumaric acid induces antioxidant capacity and defense responses of sweet cherry fruit to fungal pathogens. Postharvest Biol. Technol. 169, 111297. doi: 10.1016/j.postharvbio.2020.111297

[B37] Love M. Huber W. Anders S. (2014). Moderated estimation of fold change and dispersion for RNA-seq data with DESeq2. Genome Biol 15:550. doi: 10.1186/s13059-014-0550-8 25516281 PMC4302049

[B38] Luo W. Brouwer C. (2013). Pathview: an R/Bioconductor package for pathway-based data integration and visualization. Bioinformatics 29, 1830–1831. doi: 10.1093/bioinformatics/btt285 23740750 PMC3702256

[B39] Luypaert G. Witters J. van Huylenbroeck J. Clercq P. Riek J. De Keyser E. (2017). Induced expression of selected plant defence related genes in pot azalea, *Rhododendron simsii* hybrid. Euphytica 213, 227. doi: 10.1007/s10681-017-2010-5

[B40] Malerba M. Cerana R. (2019). Recent applications of chitin- and chitosan-based polymers in plants. Polymers 11, 839. doi: 10.3390/polym11050839 31072059 PMC6572233

[B41] Martínez G. Regente M. Jacobi S. Del Rio M. Pinedo M. de la Canal L. (2017). Chlorogenic acid is a fungicide active against phytopathogenic fungi. Pestic. Biochem. Physiol. 140, 30–35. doi: 10.1016/j.pestbp.2017.05.012 28755691

[B42] Mauch-Mani B. Baccelli I. Luna E. Flors V. (2017). Defense priming: an adaptive part of induced resistance. Annu. Rev. Plant Biol. 68, 485–512. doi: 10.1146/annurev-arplant-042916-041132 28226238

[B43] Mi H. Muruganujan A. Huang X. Ebert D. Mills C. Guo X.. (2019). Protocol update for large-scale genome and gene function analysis with the PANTHER classification system (v.14.0). Nat. Protoc 14, 703–721. doi: 10.1038/s41596-019-0128-8 30804569 PMC6519457

[B44] Moreno-Escamilla J. O. Jimeńez-Hernández F. E. Alvarez-Parrilla E. de la Rosa L. A. Martínez-Ruiz N. D. R. González-Fernández R.. (2020). Effect of elicitation on polyphenol and carotenoid metabolism in butterhead lettuce (*Lactuca sativa* var. capitata). ACS Omega 5, 11535–11546. doi: 10.1021/acsomega.0c00680 PMC7254786 32478243

[B45] Mortensen L. M. (1986). Effect of relative humidity on growth and flowering of some greenhouse plants. Sci. Hortic. 29, 301–307. doi: 10.1016/0304-4238(86)90013-0

[B46] Mou B. (2008). “Lettuce,” in Vegetables I: Asteraceae, brassicaceae, chenopodicaceae, and cucurbitaceae. Eds. Prohens J. Nuez F. (New York, NY: Springer New York), 75–116.

[B47] Muymas P. Pichyangkura R. Wiriyakitnateekul W. Wangsomboondee T. Chadchawan S. Seraypheap K. (2015). Effects of chitin-rich residues on growth and postharvest quality of lettuce. Biol. Agric. Hortic. 31, 108–117. doi: 10.1080/01448765.2014.974669

[B48] Newman M.-A. Sundelin T. Nielsen J. T. Erbs G. (2013). MAMP (microbe-associated molecular pattern) triggered immunity in plants. Front. Plant Sci. 4, 139. doi: 10.3389/fpls.2013.00139 PMC3655273 23720666

[B49] Okonechnikov K. Conesa A. García-Alcalde F. (2015). Qualimap 2: advanced multi-sample quality control for high-throughput sequencing data. Bioinformatics 32, 292–294. doi: 10.1093/bioinformatics/btv566 26428292 PMC4708105

[B50] Peng Y. Yang J. Li X. Zhang Y. (2021). Salicylic acid: biosynthesis and signaling. Annu. Rev. Plant Biol. 72, 761–791. doi: 10.1146/annurev-arplant-081320-092855 33756096

[B51] RStudio Team (2019). RStudio: Integrated development environment for R (Boston: MA: RStudio, Inc).

[B52] Savci S. (2012). An agricultural pollutant: chemical fertilizer. Int. J. Environ. Sci. Dev. 3, 73–80. doi: 10.7763/ijesd.2012.v3.191

[B53] Shamshina J. L. Kelly A. Oldham T. Rogers R. D. (2020). Agricultural uses of chitin polymers. Environ. Chem. Lett. 18, 53–60. doi: 10.1007/s10311-019-00934-5

[B54] Sharp R. (2013). A review of the applications of chitin and its derivatives in agriculture to modify plant-microbial interactions and improve crop yields. Agronomy 3, 757–793. doi: 10.3390/agronomy3040757

[B55] Subbarao K. V. Davis R. M. Gilbertson R. L. Raid R. N. (2017). Compendium of lettuce diseases and pests. 2nd ed. (St. Paul, MN: The American Phytopathological Society).

[B56] Takahashi F. Shinozaki K. (2019). Long-distance signaling in plant stress response. Curr. Opin. Plant Biol. 47, 106–111. doi: 10.1016/j.pbi.2018.10.006 30445314

[B57] Team R. C. (2020). R: A language and environment for statistical computing (Vienna, Austria: The R Project for Statistical Computing).

[B58] Tibbitts T. W. Bottenberg G. (1976). Growth of lettuce under controlled humidity levels1. J. Amer. Soc Hortic. Sci. 101, 70–73. doi: 10.21273/JASHS.101.1.70

[B59] Velásquez C. L. Pirela M. R. (2016). “Biochemical aspects of the chitin fungicidal activity in agricultural uses,” in Chitosan in the preservation of agricultural commodities. Eds. Bautista-Baños S. Romanazzi G. Jiménez-Aparicio A. (Amsterdam, Boston, Heidelberg: Academic Press), 279–298.

[B60] Wojciechowska E. Weinert C. H. Egert B. Trierweiler B. Schmidt-Heydt M. Horneburg B.. (2014). Chlorogenic acid, a metabolite identified by untargeted metabolome analysis in resistant tomatoes, inhibits the colonization by *Alternaria alternata* by inhibiting alternariol biosynthesis. Eur. J. Plant Pathol. 139, 735–747. doi: 10.1007/s10658-014-0428-3

[B61] Wu T. Hu E. Xu S. Chen M. Guo P. Dai Z.. (2021). clusterProfiler 4.0: a universal enrichment tool for interpreting omics data. Innovation (Camb.) 2, 100141. doi: 10.1016/j.xinn.2021.100141 34557778 PMC8454663

[B62] Yuan S. Li W. Li Q. Wang L. Cao J. Jiang W. (2019). Defense responses, induced by *p*-coumaric acid and methyl *p*-coumarate, of jujube (*Ziziphus jujuba* mill.) fruit against black spot rot caused by *Alternaria alternata* . J. Agric. Food. Chem. 67, 2801–2810. doi: 10.1021/acs.jafc.9b00087 30794401

[B63] Zhang D. Bi W. Kai K. Ye Y. Liu J. (2020). Effect of chlorogenic acid on controlling kiwifruit postharvest decay caused by *Diaporthe* sp. LWT 132, 109805. doi: 10.1016/j.lwt.2020.109805

