# Supplementary material for: Assessment of transcriptional reprogramming of lettuce roots in response to chitin soil amendment
Source: Front Plant Sci. 2023 Apr 5;14:1158068. doi: 10.3389/fpls.2023.1158068 (PMC10115174; doi:10.3389/fpls.2023.1158068)
Supplement: Supplementary file 1 [file DataSheet_1.docx]

Supplementary Material

belonging to

Assessment of transcriptional reprogramming of lettuce roots in response to chitin soil amendment

Leilei Li^1,2*†^, Moritz Kaufmann^3,4†^, Moffat Makechemu^4^, Christof Van Poucke^5^, Ellen De Keyser^1^, Mieke Uyttendaele^2^, Cyril Zipfel^4,6^, Bart Cottyn^1‡^ and Joël F. Pothier^3*‡^

# Supplementary Data

# Text S1. RT-qPCR of lettuce genes 48 and 72h post-transplantating.

# Supplementary Tables

**Supplementary Table 1.** Overview of differentially expressed genes (DEGs) in lettuce roots between lettuce grown in chitin-amended potting soil and lettuce grown in potting soil.

**Supplementary Table 2.** Overview of differentially expressed genes (DEGs) in lettuce leaves between lettuce grown in chitin-amended potting soil and lettuce grown in potting soil.

**Supplementary Table 3.** Overview of the reference compounds for the targeted LC-MS metabolomics analysis.

**Supplementary Table 4.** Reference genes tested for RT-qPCR.

**Supplementary Table 5.** List of primers tested for target genes.

# Supplementary Figures

**Supplementary Figure 1.** KEGG pathway of the phenylpropanoid biosynthesis pathway with differentially expressed genes mapped to it.

**Supplementary Figure 2.** KEGG pathway of the flavonoid biosynthesis pathway with differentially expressed genes mapped to it.

**Supplementary Figure 3.** KEGG pathway of the photosynthesis pathway with differentially expressed genes mapped to it.

**Supplementary Figure 4.** Principal component analysis plot of untargeted method on the LC-HRMS in both positive ionization (ESI pos) and negative ionization (ESI neg).

**Supplementary Figure 5.** Virtual gel electrophoresis and RNA integrity number evaluated using BioAnalyzer.

**Supplementary Figure 6.** Relative gene expression profiles in both leaf and root tissues obtained by RT-qPCR (CNQR not log transformed).

# Supplementary Data

# Text S1. RT-qPCR of lettuce genes 48 and 72h post-transplanting.

After RNA extraction using the CTAB method (Luypaert et al. 2017), the yield and purity of the obtained RNA were measured using a Nanodrop spectrophotometer (Isogen Life Sciences, Utrecht, Netherlands). The integrity was determined on a representative subset of the samples using a BioAnalyzer (Agilent technologies, Santa Clara, CA, USA). RNA integrity (RIN) was evaluated based on the clear ribosomal bands in the virtual gel view together with the RIN value (>7) (**Supplementary Figure 5**). Contaminating DNA was removed using DNA-*free* DNA removal kit (AM1906, Invitrogen; Thermo Fisher Scientific, Waltham, MA, USA) according to the manufacturer’s protocol.

For cDNA synthesis, the starting amount of RNA was adjusted to 1,000 ng in 10 µL volume. All samples were prepared in double, one for reverse transcription (RT) and one for noRT, respectively. iScript cDNA synthesis kit (Bio-Rad, Schiltigheim, France) was used for cDNA synthesis. A mix of 4 µL of 5× iScript reaction mix, 5 µL of nuclease-free water and 1 µL iScript reverse transcriptase were added to the RT sample. For noRT samples, 10 µL of nuclease-free water was added to the cDNA. RT and noRT samples were placed in a PCR machine for 5 min 25℃, 20 min 46℃, 1 min 95℃.

For the RT-qPCR, 2 µL of cDNA or noRT sample was added to a mixture of 300 nM of both forward and reverse primer and 1× SensiFAST SYBR No-ROX Mix (Bioline, Luckenwalde, Germany). Real-time detection (including melting curve analysis) was performed in a LightCycler480 (Roche, Basel, Switzerland) as described in De Keyser et al. (2020). No template controls (NTC) were also added.

Reference genes were selected out of eight genes (**Supplementary Table 4**) that have been tested in lettuce before (Borowski et al., 2014). Based on the GeNorm assay, two genes, *APT1* and *TIP41*, were selected for calculation of the normalization factor (*M* = 0.296; *CV* = 0.103). Primers for target genes (**Supplementary Table 5**) were designed using Primer3plus software, with length of ~20 bp, *T_m_* of ~60℃ and product length ~200 bp (Untergasser et al., 2007). Amplification efficiency was calculated based on the amplification curves of all samples using LinReg PCR software (Untergasser et al., 2021). Calculation of relative gene expression was done in qbase+ software (Biogazelle, Zwijnaarde, Belgium). CNRQ values (scaled to average) were exported to MS Excel.

In total 38 target genes were tested, 14 genes were actually expressed. The rest showed a *C_q_* value above 35 and were discarded for further analysis. The calculated normalized relative quantity (CNRQ) of the different genes is shown in **Supplementary Figure 6**. Statistical analysis (Kruskal-Wallis test) showed no difference between different samples for each target gene. However, *AOS* is highly expressed in root, and is slightly higher in chitin-treated samples at 72 hpt compared to control. Previous research has revealed that AOS-derived oxylipins control the links between growth and defense (Farmer and Goossens, 2019, Sivasankar et al., 2000). In root, the expression of *MYB 13*, a transcription factor regulating flavonoid biosynthesis, was also slightly higher in the chitin-treated group compared to the control.

# Supplementary Tables:

**Supplementary Table 1.** Overview of significant different expressed genes (DEGs) compared between lettuce roots grown in chitin-amended potting soil and potting soil. The first column shows the locus tag of the corresponding gene in the genome of *Lactuca sativa* cv. ‘Salinas’. Column two to five show the base mean of the gene the log_2_-fold change of these gene in the chitin-treated condition, the standard error of the log_2_-fold change estimate (lfcSE), the *p*-value and the adjusted *p*-value.

| **Locus tag** | **Base mean** | **log_2_-fold change** | **lfcSE** | ***p-*value** | ***p-*adj** |
| --- | --- | --- | --- | --- | --- |
| LSAT_2X42860 | 18.3971569 | 6.86254053 | 1.56265456 | 2.25E-08 | 2.54E-06 |
| LSAT_8X66261 | 95.5421457 | 6.47810574 | 1.04674659 | 3.68E-11 | 1.52E-08 |
| LSAT_6X76240 | 184.061841 | 5.72565134 | 1.14953335 | 8.82E-09 | 1.24E-06 |
| LSAT_9X181 | 5.92627821 | 5.1616563 | 1.76542748 | 4.82E-07 | 2.89E-05 |
| LSAT_2X77261 | 23.0923129 | 5.01862526 | 1.40803752 | 1.76E-07 | 1.34E-05 |
| LSAT_2X76880 | 178.559334 | 4.69046385 | 0.88267231 | 1.20E-09 | 2.61E-07 |
| LSAT_6X18120 | 707.366554 | 4.65226725 | 1.09285919 | 3.11E-08 | 3.26E-06 |
| LSAT_3X2981 | 208.150384 | 4.62263367 | 1.39558363 | 2.49E-07 | 1.76E-05 |
| LSAT_6X76700 | 99.479962 | 4.13224054 | 1.75293546 | 1.33E-06 | 6.47E-05 |
| LSAT_4X172320 | 22.8313033 | 4.04716851 | 1.05869989 | 4.11E-08 | 4.02E-06 |
| LSAT_3X102861 | 129.987847 | 3.66642242 | 1.51512865 | 1.08E-06 | 5.55E-05 |
| LSAT_6X76441 | 158.765684 | 3.59350738 | 1.00838329 | 3.88E-08 | 3.88E-06 |
| LSAT_3X59241 | 1775.36816 | 3.07151004 | 1.09966457 | 2.77E-07 | 1.91E-05 |
| LSAT_6X32141 | 747.624172 | 3.02445946 | 1.61944449 | 3.69E-06 | 0.00014239 |
| LSAT_4X28860 | 33634.0432 | 2.75848784 | 0.87187439 | 6.10E-08 | 5.59E-06 |
| LSAT_9X81040 | 1452.81289 | 2.73405633 | 1.26118733 | 1.80E-06 | 8.20E-05 |
| LSAT_9X97280 | 11.5664012 | 2.7240075 | 1.25101699 | 1.76E-06 | 8.07E-05 |
| LSAT_8X77040 | 387.615324 | 2.69594612 | 1.64769497 | 6.85E-06 | 0.00023039 |
| LSAT_6X73360 | 4038.27096 | 2.6927477 | 1.00102741 | 3.72E-07 | 2.34E-05 |
| LSAT_5X112041 | 89.4322274 | 2.55419683 | 1.46994696 | 5.78E-06 | 0.00019901 |
| LSAT_3X141021 | 6141.13655 | 2.54992482 | 0.73219686 | 1.85E-08 | 2.25E-06 |
| LSAT_3X91921 | 166.858483 | 2.26159157 | 0.79751974 | 4.36E-07 | 2.68E-05 |
| LSAT_6X97181 | 2907.16285 | 2.22508829 | 0.79474928 | 5.37E-07 | 3.11E-05 |
| LSAT_2X2161 | 2118.77077 | 2.19797926 | 0.86555437 | 1.18E-06 | 5.91E-05 |
| LSAT_6X9740 | 184.444247 | 2.19550545 | 1.08978019 | 4.19E-06 | 0.00015652 |
| LSAT_9X102320 | 939.49444 | 2.09473159 | 0.76140397 | 9.17E-07 | 4.89E-05 |
| LSAT_5X6120 | 2041.63712 | 1.95379628 | 1.404224 | 2.02E-05 | 0.00053157 |
| LSAT_4X164620 | 1099.97471 | 1.84986893 | 1.02327868 | 1.19E-05 | 0.00035255 |
| LSAT_2X44620 | 10.3366542 | 1.80821347 | 1.59270581 | 3.67E-05 | 0.00083714 |
| LSAT_7X51761 | 734.055414 | 1.79009607 | 0.61222043 | 2.14E-06 | 9.36E-05 |
| LSAT_9X117441 | 14818.6169 | 1.75836411 | 0.77094412 | 7.33E-06 | 0.0002414 |
| LSAT_4X26101 | 850.368112 | 1.75569379 | 0.89125354 | 1.18E-05 | 0.00034997 |
| LSAT_8X11480 | 139.201328 | 1.74348837 | 0.81892979 | 9.69E-06 | 0.00030041 |
| LSAT_5X46341 | 26405.6755 | 1.74268391 | 0.60927201 | 2.98E-06 | 0.00011965 |
| LSAT_7X84560 | 637.308969 | 1.74211697 | 0.93933095 | 1.44E-05 | 0.00040967 |
| LSAT_6X78800 | 6134.97463 | 1.66981852 | 0.32604199 | 5.13E-09 | 8.50E-07 |
| LSAT_9X109041 | 17.095728 | 1.66666951 | 0.43801042 | 5.46E-07 | 3.14E-05 |
| LSAT_0X5541 | 202.960203 | 1.65816785 | 0.45158062 | 7.98E-07 | 4.33E-05 |
| LSAT_4X84020 | 4880.34828 | 1.62148184 | 0.71208608 | 1.22E-05 | 0.00035696 |
| LSAT_5X181161 | 4368.02277 | 1.5911114 | 0.38996937 | 3.45E-07 | 2.23E-05 |
| LSAT_4X28820 | 17810.7298 | 1.56841725 | 0.61303864 | 1.02E-05 | 0.00031367 |
| LSAT_6X74860 | 720.794406 | 1.56718294 | 0.50228865 | 3.94E-06 | 0.00014945 |
| LSAT_7X34960 | 18852.2788 | 1.55116802 | 0.55877142 | 7.80E-06 | 0.00025314 |
| LSAT_8X60981 | 53580.1631 | 1.52647994 | 0.26825976 | 5.32E-10 | 1.36E-07 |
| LSAT_5X23101 | 468.427926 | 1.51804313 | 0.67188557 | 1.87E-05 | 0.00050224 |
| LSAT_9X29121 | 1523.0227 | 1.47281346 | 0.69909205 | 2.66E-05 | 0.00064894 |
| LSAT_3X47940 | 519.538436 | 1.46769166 | 0.2630967 | 1.06E-09 | 2.39E-07 |
| LSAT_6X78520 | 593.11486 | 1.44184395 | 0.84642768 | 4.32E-05 | 0.0009488 |
| LSAT_3X73300 | 50653.429 | 1.4411532 | 0.65826785 | 2.76E-05 | 0.00066895 |
| LSAT_6X81741 | 4048.78074 | 1.3949191 | 0.45458225 | 7.63E-06 | 0.00024841 |
| LSAT_9X54780 | 152.930546 | 1.39403936 | 1.01189704 | 6.32E-05 | 0.0012675 |
| LSAT_6X110001 | 1269.75864 | 1.38646294 | 0.57944844 | 2.66E-05 | 0.00064894 |
| LSAT_5X125561 | 12973.7504 | 1.3808883 | 0.5806833 | 2.77E-05 | 0.00066959 |
| LSAT_3X41120 | 352.483261 | 1.37516607 | 0.68876376 | 4.42E-05 | 0.00096332 |
| LSAT_8X13541 | 3130.60924 | 1.3466443 | 0.91057617 | 6.96E-05 | 0.00136397 |
| LSAT_8X160040 | 548.196664 | 1.343705 | 0.28043211 | 3.88E-08 | 3.88E-06 |
| LSAT_2X24040 | 4406.43572 | 1.33462561 | 0.55214174 | 3.10E-05 | 0.00073124 |
| LSAT_5X142940 | 15.4272014 | 1.32851463 | 1.03456362 | 8.09E-05 | 0.00152615 |
| LSAT_6X14640 | 60.9058472 | 1.29471272 | 0.25794338 | 1.38E-08 | 1.81E-06 |
| LSAT_7X47621 | 3949.25684 | 1.28402779 | 0.35237966 | 2.00E-06 | 8.86E-05 |
| LSAT_8X110401 | 519.643738 | 1.27478705 | 0.36597597 | 3.18E-06 | 0.00012589 |
| LSAT_4X60501 | 157.828494 | 1.27424149 | 0.74957029 | 8.33E-05 | 0.00155777 |
| LSAT_5X103641 | 1254.92142 | 1.25253877 | 0.73347947 | 9.12E-05 | 0.00167299 |
| LSAT_1X35440 | 438.417089 | 1.25174653 | 0.2936148 | 2.57E-07 | 1.80E-05 |
| LSAT_8X154721 | 957.148702 | 1.24445159 | 0.43380414 | 1.59E-05 | 0.00044121 |
| LSAT_8X42980 | 12.2035057 | 1.24338642 | 0.48653094 | 3.16E-05 | 0.00073974 |
| LSAT_3X41281 | 35.9022296 | 1.19919731 | 0.74068984 | 0.00011954 | 0.00207598 |
| LSAT_8X43780 | 103.693393 | 1.18617517 | 0.31487734 | 1.26E-06 | 6.23E-05 |
| LSAT_5X186721 | 41883.2566 | 1.18213792 | 0.48387648 | 4.55E-05 | 0.00098635 |
| LSAT_7X83841 | 105.698484 | 1.18165143 | 0.1875263 | 1.17E-11 | 5.87E-09 |
| LSAT_9X92240 | 188.120631 | 1.16937119 | 0.6574009 | 0.00012674 | 0.00215276 |
| LSAT_8X136401 | 609.097548 | 1.16812106 | 0.57944509 | 9.98E-05 | 0.00179535 |
| LSAT_3X139861 | 8229.94815 | 1.15350761 | 0.24246353 | 2.74E-08 | 2.96E-06 |
| LSAT_8X59160 | 855.644215 | 1.15039675 | 0.25582085 | 8.01E-08 | 7.02E-06 |
| LSAT_4X99780 | 170.29596 | 1.13988442 | 0.48639557 | 6.13E-05 | 0.00124732 |
| LSAT_4X130161 | 228.217374 | 1.13839266 | 0.62934266 | 0.00014162 | 0.00234892 |
| LSAT_6X35480 | 24122.5897 | 1.1271182 | 0.53490875 | 9.95E-05 | 0.00179224 |
| LSAT_7X83821 | 56.9357805 | 1.12400723 | 0.26801224 | 2.40E-07 | 1.72E-05 |
| LSAT_8X98481 | 38336.9293 | 1.11783935 | 0.70985886 | 0.00017617 | 0.0027563 |
| LSAT_2X21801 | 26.7896072 | 1.10450944 | 0.2945058 | 1.14E-06 | 5.75E-05 |
| LSAT_2X111580 | 20.2055008 | 1.10422375 | 0.40826036 | 2.75E-05 | 0.0006666 |
| LSAT_9X74980 | 4918.46294 | 1.10143323 | 0.44510124 | 4.91E-05 | 0.0010447 |
| LSAT_1X83120 | 57.3511824 | 1.09799567 | 0.4042939 | 2.68E-05 | 0.00065253 |
| LSAT_3X52120 | 11.0588151 | 1.09646994 | 0.27435949 | 4.60E-07 | 2.80E-05 |
| LSAT_2X59820 | 1479.99307 | 1.09456047 | 0.14284428 | 6.59E-16 | 2.86E-12 |
| LSAT_6X16401 | 1481.32873 | 1.08725641 | 0.39854951 | 2.61E-05 | 0.00064099 |
| LSAT_3X40600 | 57.7810169 | 1.07900857 | 0.37504863 | 1.74E-05 | 0.00047293 |
| LSAT_5X6580 | 3337.96031 | 1.06973846 | 0.2935031 | 1.52E-06 | 7.17E-05 |
| LSAT_1X35241 | 14714.7359 | 1.06657893 | 0.36969562 | 1.70E-05 | 0.00046419 |
| LSAT_2X47140 | 744.118409 | 1.06498394 | 0.48831403 | 0.00010158 | 0.0018194 |
| LSAT_4X97140 | 402.633828 | 1.06255148 | 1.14585835 | 0.0001904 | 0.00291436 |
| LSAT_1X112881 | 15.3945978 | 1.06239234 | 1.27856782 | 0.00018606 | 0.00286572 |
| LSAT_4X360 | 817.869069 | 1.05848317 | 0.48940421 | 0.00010708 | 0.0019038 |
| LSAT_2X102440 | 119.700121 | 1.05143002 | 0.50578062 | 0.00012923 | 0.00218426 |
| LSAT_7X86661 | 12.4543143 | 1.04728943 | 0.33709736 | 8.63E-06 | 0.00027449 |
| LSAT_9X12001 | 17366.4441 | 1.04428233 | 0.48364459 | 0.00011139 | 0.0019657 |
| LSAT_3X1560 | 593.251945 | 1.04298394 | 0.2825158 | 1.20E-06 | 6.00E-05 |
| LSAT_2X84540 | 227.075749 | 1.03817647 | 0.22184396 | 2.19E-08 | 2.49E-06 |
| LSAT_8X152120 | 5142.15278 | 1.02806716 | 0.43259763 | 6.89E-05 | 0.00135561 |
| LSAT_2X133560 | 79.2179844 | 1.01812473 | 0.26294119 | 5.71E-07 | 3.27E-05 |
| LSAT_8X86420 | 118.018339 | 1.01308411 | 0.20184728 | 3.85E-09 | 6.73E-07 |
| LSAT_9X29301 | 69.4482133 | 1.00884584 | 0.23733023 | 1.21E-07 | 9.91E-06 |
| LSAT_1X27760 | 192.452888 | 1.0073005 | 1.07624005 | 0.000229 | 0.00334935 |
| LSAT_6X21140 | 2341.34826 | 1.0063903 | 0.29667955 | 3.19E-06 | 0.00012606 |
| LSAT_4X113481 | 29.0925562 | 1.00560473 | 0.17573451 | 9.21E-11 | 3.20E-08 |
| LSAT_8X1141 | 47.018 | 1.00438114 | 0.23755074 | 1.31E-07 | 1.06E-05 |
| LSAT_7X114581 | 268.39418 | 1.00167096 | 0.23741941 | 1.34E-07 | 1.08E-05 |
| LSAT_4X173760 | 21.6779166 | -1.00057264 | 0.59783965 | 0.00028725 | 0.00393043 |
| LSAT_1X31461 | 22.2531005 | -1.00083643 | 0.34993878 | 1.80E-05 | 0.00048827 |
| LSAT_2X89180 | 14.3792979 | -1.00299114 | 0.62984432 | 0.000303 | 0.00409703 |
| LSAT_7X28721 | 5.0952857 | -1.00409061 | 0.48587709 | 0.00014878 | 0.00242914 |
| LSAT_3X30521 | 5790.78656 | -1.00447547 | 0.30048429 | 3.77E-06 | 0.00014443 |
| LSAT_1X118261 | 438.209521 | -1.00448427 | 0.22686191 | 5.59E-08 | 5.22E-06 |
| LSAT_8X82500 | 177.922691 | -1.00519626 | 0.1519937 | 4.22E-13 | 5.96E-10 |
| LSAT_8X81900 | 138.014449 | -1.00797922 | 0.25044749 | 3.04E-07 | 2.03E-05 |
| LSAT_2X54380 | 49.264375 | -1.01070097 | 0.27231715 | 1.02E-06 | 5.30E-05 |
| LSAT_5X12800 | 1131.95189 | -1.0117512 | 0.16032169 | 3.06E-12 | 2.45E-09 |
| LSAT_9X114860 | 46.2783893 | -1.01290026 | 0.24676284 | 2.25E-07 | 1.64E-05 |
| LSAT_8X132500 | 58.8497876 | -1.01586801 | 0.32365387 | 7.57E-06 | 0.0002467 |
| LSAT_8X59300 | 7.73161853 | -1.01728555 | 0.63907601 | 0.00028355 | 0.00389324 |
| LSAT_2X11060 | 41.4600463 | -1.01771465 | 0.44845891 | 9.11E-05 | 0.00167299 |
| LSAT_5X175381 | 431.710774 | -1.01900487 | 0.21389402 | 1.34E-08 | 1.78E-06 |
| LSAT_2X83520 | 6.05541674 | -1.01990559 | 0.49961342 | 0.00015089 | 0.00245687 |
| LSAT_2X106580 | 284.076182 | -1.02049832 | 0.39724784 | 4.18E-05 | 0.00092684 |
| LSAT_9X100640 | 20.3553288 | -1.0208383 | 0.51751311 | 0.00017212 | 0.00271066 |
| LSAT_2X89421 | 134.684163 | -1.02200242 | 0.21870653 | 2.07E-08 | 2.40E-06 |
| LSAT_4X132460 | 121.252878 | -1.02210809 | 0.25419607 | 3.25E-07 | 2.13E-05 |
| LSAT_5X132120 | 80.9816771 | -1.0224037 | 0.2779236 | 1.19E-06 | 5.97E-05 |
| LSAT_5X172161 | 39.1426211 | -1.02336973 | 0.34892613 | 1.45E-05 | 0.00041161 |
| LSAT_6X29540 | 362.945567 | -1.02748589 | 0.1755977 | 5.35E-11 | 2.07E-08 |
| LSAT_2X30320 | 15.723579 | -1.02881909 | 0.35146787 | 1.48E-05 | 0.00041634 |
| LSAT_4X54361 | 66.0045867 | -1.02916903 | 0.31590448 | 5.21E-06 | 0.00018447 |
| LSAT_5X19781 | 34.1680079 | -1.02977809 | 0.31117992 | 4.40E-06 | 0.00016216 |
| LSAT_9X106200 | 3.58171609 | -1.03068774 | 0.93242258 | 0.00022934 | 0.00335221 |
| LSAT_6X66601 | 9.97790524 | -1.03125086 | 0.47911765 | 0.00011628 | 0.00203116 |
| LSAT_7X66640 | 72.1788324 | -1.03157159 | 0.27702373 | 1.04E-06 | 5.36E-05 |
| LSAT_5X123580 | 11.3191836 | -1.03341668 | 0.49518445 | 0.00013297 | 0.0022339 |
| LSAT_8X28421 | 196.030871 | -1.03592462 | 0.22382649 | 2.72E-08 | 2.96E-06 |
| LSAT_4X82580 | 82.9938189 | -1.03616528 | 0.25253298 | 2.48E-07 | 1.75E-05 |
| LSAT_8X147461 | 909.03277 | -1.03718752 | 0.23803789 | 8.83E-08 | 7.51E-06 |
| LSAT_5X13860 | 719.885061 | -1.04036124 | 0.16178719 | 1.90E-12 | 1.80E-09 |
| LSAT_2X97521 | 27.3578016 | -1.04181643 | 0.3421128 | 1.04E-05 | 0.00031905 |
| LSAT_7X47340 | 229.88034 | -1.04690187 | 0.21133764 | 6.52E-09 | 1.01E-06 |
| LSAT_1X3920 | 7.68377561 | -1.0474973 | 0.54193662 | 0.00017039 | 0.00269741 |
| LSAT_5X77661 | 294.276791 | -1.04993166 | 0.22511513 | 2.50E-08 | 2.74E-06 |
| LSAT_4X111181 | 18.7383786 | -1.05056299 | 0.60703854 | 0.00022041 | 0.00325142 |
| LSAT_4X107341 | 61.3856133 | -1.05218863 | 0.4937477 | 0.00011609 | 0.00203077 |
| LSAT_6X20120 | 432.839884 | -1.05880861 | 0.20639449 | 3.02E-09 | 5.61E-07 |
| LSAT_8X52240 | 13.989635 | -1.06064918 | 0.46591422 | 8.32E-05 | 0.00155553 |
| LSAT_2X76100 | 592.578976 | -1.06153851 | 0.17258101 | 1.25E-11 | 6.01E-09 |
| LSAT_4X167900 | 36.8285849 | -1.06211213 | 0.26983014 | 5.17E-07 | 3.06E-05 |
| LSAT_4X65440 | 5.14621485 | -1.0621137 | 0.51489815 | 0.0001296 | 0.00218624 |
| LSAT_8X153541 | 38.0451231 | -1.06323749 | 0.34284403 | 8.90E-06 | 0.00028175 |
| LSAT_6X79821 | 2659.92937 | -1.0657188 | 0.25027595 | 1.50E-07 | 1.18E-05 |
| LSAT_5X117961 | 3690.46663 | -1.06828924 | 0.21574584 | 7.49E-09 | 1.13E-06 |
| LSAT_7X13080 | 17.6843358 | -1.07255372 | 0.51133211 | 0.00011795 | 0.00205292 |
| LSAT_5X172141 | 51.431128 | -1.07322743 | 0.38442047 | 2.21E-05 | 0.00056797 |
| LSAT_8X84940 | 396.328755 | -1.07357163 | 0.18509672 | 1.03E-10 | 3.47E-08 |
| LSAT_8X147700 | 747.41268 | -1.07451598 | 0.22140547 | 1.22E-08 | 1.63E-06 |
| LSAT_8X2701 | 352.70325 | -1.07455415 | 0.14508071 | 3.63E-15 | 8.72E-12 |
| LSAT_5X170741 | 26120.6963 | -1.07883464 | 0.29989086 | 1.82E-06 | 8.27E-05 |
| LSAT_5X132941 | 278.81932 | -1.0790028 | 0.18560064 | 9.93E-11 | 3.40E-08 |
| LSAT_9X81480 | 85.3922777 | -1.07928659 | 0.23853273 | 5.30E-08 | 5.03E-06 |
| LSAT_2X24081 | 18.0241455 | -1.07981914 | 0.38841828 | 2.28E-05 | 0.0005826 |
| LSAT_3X24581 | 15.6290785 | -1.08108005 | 0.41083342 | 3.39E-05 | 0.00078392 |
| LSAT_7X83921 | 71.8442235 | -1.0835964 | 0.27426645 | 5.24E-07 | 3.08E-05 |
| LSAT_1X71780 | 34.0318986 | -1.08598319 | 0.39329323 | 2.39E-05 | 0.00060296 |
| LSAT_8X114181 | 58.5401491 | -1.08724115 | 0.52886877 | 0.00012233 | 0.00210471 |
| LSAT_8X32181 | 9.32589343 | -1.09642328 | 0.48618798 | 8.11E-05 | 0.00152729 |
| LSAT_8X133941 | 22.9208967 | -1.09881335 | 0.25625762 | 1.52E-07 | 1.19E-05 |
| LSAT_0X2801 | 930.61079 | -1.10394485 | 0.207405 | 1.55E-09 | 3.27E-07 |
| LSAT_6X58140 | 67.8342154 | -1.10440952 | 0.33547118 | 5.13E-06 | 0.00018174 |
| LSAT_5X171780 | 8.42050367 | -1.10488222 | 0.38491915 | 1.76E-05 | 0.00047836 |
| LSAT_4X99701 | 518.169276 | -1.1056406 | 0.23372118 | 2.48E-08 | 2.73E-06 |
| LSAT_4X120941 | 398.480948 | -1.10771743 | 0.2894655 | 8.78E-07 | 4.70E-05 |
| LSAT_4X109800 | 53.5382812 | -1.11140511 | 0.21605687 | 3.87E-09 | 6.73E-07 |
| LSAT_6X99040 | 62.2756897 | -1.11264265 | 0.2688279 | 2.84E-07 | 1.93E-05 |
| LSAT_6X80160 | 84.534448 | -1.11683167 | 0.32552032 | 3.37E-06 | 0.00013242 |
| LSAT_2X19960 | 309.399444 | -1.11897311 | 0.2057105 | 9.44E-10 | 2.18E-07 |
| LSAT_5X91281 | 22.4932211 | -1.12097066 | 0.41081142 | 2.56E-05 | 0.00063092 |
| LSAT_1X89881 | 47.1888773 | -1.12128683 | 0.36931167 | 1.11E-05 | 0.00033386 |
| LSAT_8X131640 | 63.6108405 | -1.12235836 | 0.22380226 | 7.53E-09 | 1.13E-06 |
| LSAT_9X30661 | 6.65261484 | -1.12369372 | 0.55243068 | 0.00011334 | 0.00199579 |
| LSAT_3X139941 | 42.9964042 | -1.12894419 | 0.47164764 | 5.64E-05 | 0.00116219 |
| LSAT_9X50060 | 774.183211 | -1.13023623 | 0.1838469 | 2.14E-11 | 9.33E-09 |
| LSAT_8X140400 | 14.507914 | -1.13053614 | 0.389215 | 1.60E-05 | 0.00044193 |
| LSAT_4X120701 | 622.042941 | -1.13338493 | 0.17003287 | 9.18E-13 | 1.05E-09 |
| LSAT_3X6741 | 288.86496 | -1.1335307 | 0.18502623 | 2.47E-11 | 1.06E-08 |
| LSAT_3X21901 | 51.0643636 | -1.13470099 | 0.45006659 | 4.19E-05 | 0.00092684 |
| LSAT_4X37601 | 144.845596 | -1.1357251 | 0.18991504 | 5.79E-11 | 2.21E-08 |
| LSAT_7X34841 | 20.7281538 | -1.13917159 | 0.37383232 | 1.08E-05 | 0.00032707 |
| LSAT_6X69600 | 164.353804 | -1.1417155 | 0.23003698 | 1.05E-08 | 1.44E-06 |
| LSAT_5X175680 | 56.2080043 | -1.14284018 | 0.26859419 | 2.02E-07 | 1.50E-05 |
| LSAT_6X38501 | 18.1962404 | -1.14718246 | 0.52420055 | 8.14E-05 | 0.00153159 |
| LSAT_2X96161 | 395.79077 | -1.14926522 | 0.22227934 | 4.17E-09 | 7.05E-07 |
| LSAT_1X121241 | 18.8109743 | -1.15185442 | 0.29722496 | 8.22E-07 | 4.45E-05 |
| LSAT_8X14400 | 525.715474 | -1.15187969 | 0.24263465 | 2.84E-08 | 3.03E-06 |
| LSAT_1X116040 | 39.6775073 | -1.15429007 | 0.34320867 | 4.32E-06 | 0.00016005 |
| LSAT_9X88301 | 888.495216 | -1.15806177 | 0.20173024 | 2.47E-10 | 7.31E-08 |
| LSAT_1X5460 | 502.39516 | -1.15873517 | 0.21941709 | 2.58E-09 | 4.98E-07 |
| LSAT_4X134060 | 90.7377668 | -1.16321167 | 0.28953184 | 5.13E-07 | 3.05E-05 |
| LSAT_0X20741 | 130.729876 | -1.1755596 | 0.16853898 | 1.57E-13 | 2.51E-10 |
| LSAT_8X23801 | 19.4466275 | -1.17694557 | 0.41760808 | 1.94E-05 | 0.00051606 |
| LSAT_7X28400 | 127.184312 | -1.17808315 | 0.28082041 | 2.80E-07 | 1.92E-05 |
| LSAT_4X124880 | 478.549942 | -1.18074676 | 0.15672816 | 3.14E-15 | 8.39E-12 |
| LSAT_8X2021 | 30.833697 | -1.18652846 | 1.19321447 | 0.00013317 | 0.0022339 |
| LSAT_5X50940 | 7.69747013 | -1.18947159 | 0.49629763 | 4.92E-05 | 0.00104627 |
| LSAT_2X15261 | 69.1555118 | -1.19480348 | 0.27188125 | 1.38E-07 | 1.10E-05 |
| LSAT_6X4301 | 61.0296283 | -1.19481717 | 0.27711832 | 1.90E-07 | 1.43E-05 |
| LSAT_2X2780 | 36.6103716 | -1.19676359 | 0.35342514 | 4.15E-06 | 0.00015529 |
| LSAT_9X88241 | 91.9163894 | -1.20067309 | 0.48885623 | 4.25E-05 | 0.00093861 |
| LSAT_9X88221 | 126.144311 | -1.20249171 | 0.42774329 | 1.92E-05 | 0.00051428 |
| LSAT_4X54301 | 47.5164743 | -1.20623561 | 0.45719658 | 2.86E-05 | 0.00068629 |
| LSAT_6X85320 | 7.99061227 | -1.2071009 | 0.32419639 | 1.49E-06 | 7.10E-05 |
| LSAT_9X66680 | 29.9650613 | -1.20720869 | 0.49734256 | 4.44E-05 | 0.00096715 |
| LSAT_5X124661 | 13.8400243 | -1.21033104 | 0.44945449 | 2.51E-05 | 0.00062312 |
| LSAT_7X47280 | 30.6180942 | -1.22255571 | 0.42200961 | 1.53E-05 | 0.00042647 |
| LSAT_3X91640 | 300.575153 | -1.22300551 | 0.23671608 | 5.81E-09 | 9.35E-07 |
| LSAT_9X97401 | 28.9633634 | -1.2258026 | 0.47578462 | 3.14E-05 | 0.00073974 |
| LSAT_7X106841 | 4.84074223 | -1.22788811 | 0.65240369 | 9.08E-05 | 0.00167021 |
| LSAT_4X84241 | 563.844675 | -1.2301268 | 0.24394036 | 1.05E-08 | 1.44E-06 |
| LSAT_6X57741 | 221.983801 | -1.23234303 | 0.28744711 | 2.27E-07 | 1.65E-05 |
| LSAT_8X33141 | 19.6377168 | -1.23737047 | 0.24975094 | 1.58E-08 | 1.99E-06 |
| LSAT_0X16380 | 90.3047636 | -1.24077716 | 0.21948146 | 5.67E-10 | 1.40E-07 |
| LSAT_8X147680 | 40.2694834 | -1.24576105 | 0.24551474 | 9.55E-09 | 1.33E-06 |
| LSAT_9X42261 | 911.310993 | -1.25111953 | 0.2653848 | 4.54E-08 | 4.36E-06 |
| LSAT_8X51160 | 117.505154 | -1.2563378 | 0.36985797 | 4.06E-06 | 0.00015232 |
| LSAT_1X70360 | 84.5734821 | -1.25714626 | 0.24791996 | 1.00E-08 | 1.39E-06 |
| LSAT_6X60620 | 43.883553 | -1.25735399 | 0.35258749 | 2.50E-06 | 0.0001047 |
| LSAT_7X102760 | 142.429966 | -1.25775484 | 0.31960062 | 7.98E-07 | 4.33E-05 |
| LSAT_7X82221 | 100.436296 | -1.25888588 | 0.28091557 | 1.16E-07 | 9.56E-06 |
| LSAT_1X73221 | 54.5301571 | -1.25959561 | 0.41632508 | 1.07E-05 | 0.00032627 |
| LSAT_8X87921 | 100.027587 | -1.26237158 | 0.18461171 | 5.63E-13 | 7.11E-10 |
| LSAT_1X37061 | 16.5960866 | -1.26273751 | 0.49426738 | 3.02E-05 | 0.00071698 |
| LSAT_7X49380 | 55.4998982 | -1.26625403 | 0.46320531 | 2.07E-05 | 0.00054318 |
| LSAT_2X24780 | 79.7006101 | -1.26661438 | 0.28302497 | 1.20E-07 | 9.90E-06 |
| LSAT_4X185720 | 7.08153976 | -1.27333378 | 0.42637908 | 1.16E-05 | 0.00034502 |
| LSAT_3X45820 | 54.0481347 | -1.27491479 | 0.24430225 | 5.34E-09 | 8.72E-07 |
| LSAT_6X90461 | 122.875658 | -1.27692596 | 0.2075884 | 4.13E-11 | 1.68E-08 |
| LSAT_2X98701 | 213.738671 | -1.27719386 | 0.1489454 | 1.13E-18 | 2.71E-14 |
| LSAT_6X114421 | 60.8013369 | -1.27884492 | 0.31482413 | 5.36E-07 | 3.11E-05 |
| LSAT_0X10080 | 1728.64885 | -1.28077031 | 0.27177356 | 4.88E-08 | 4.66E-06 |
| LSAT_1X111020 | 1985.13036 | -1.28202316 | 0.27965645 | 8.14E-08 | 7.08E-06 |
| LSAT_2X83321 | 66.5754651 | -1.28285289 | 0.29352458 | 1.83E-07 | 1.38E-05 |
| LSAT_3X14260 | 22.8974735 | -1.2840242 | 0.36387648 | 2.79E-06 | 0.0001141 |
| LSAT_5X58101 | 21.8977088 | -1.29166333 | 0.30973468 | 3.76E-07 | 2.36E-05 |
| LSAT_1X46340 | 44.2199398 | -1.29647583 | 0.38502253 | 4.33E-06 | 0.00016006 |
| LSAT_1X64060 | 50.3464227 | -1.3039366 | 0.22092253 | 1.79E-10 | 5.44E-08 |
| LSAT_8X134220 | 104.169635 | -1.30455957 | 0.41373252 | 7.43E-06 | 0.00024348 |
| LSAT_1X95860 | 224.857128 | -1.30742289 | 0.29497524 | 1.50E-07 | 1.18E-05 |
| LSAT_8X115421 | 21.8377228 | -1.30792301 | 0.33614767 | 9.51E-07 | 5.02E-05 |
| LSAT_3X76540 | 53.2244764 | -1.3087951 | 0.26325594 | 1.74E-08 | 2.15E-06 |
| LSAT_3X44541 | 13.3817355 | -1.31165022 | 0.43371204 | 9.99E-06 | 0.00030901 |
| LSAT_7X45781 | 14.0165466 | -1.31427321 | 0.42467277 | 8.43E-06 | 0.00026879 |
| LSAT_7X10841 | 4.86145423 | -1.32765245 | 0.53914518 | 2.95E-05 | 0.00070387 |
| LSAT_0X19261 | 273.168417 | -1.33421962 | 0.23404719 | 5.57E-10 | 1.39E-07 |
| LSAT_6X83340 | 1599.78186 | -1.33468535 | 0.2503441 | 3.53E-09 | 6.47E-07 |
| LSAT_9X98681 | 236.481576 | -1.33469592 | 0.24368747 | 1.74E-09 | 3.60E-07 |
| LSAT_4X80040 | 175.5866 | -1.33531903 | 0.19784429 | 1.16E-12 | 1.26E-09 |
| LSAT_4X59521 | 695.346716 | -1.34443033 | 0.18676612 | 5.52E-14 | 1.02E-10 |
| LSAT_5X173681 | 80.2235199 | -1.34444045 | 0.37231967 | 2.16E-06 | 9.39E-05 |
| LSAT_7X100781 | 586.50556 | -1.35208207 | 0.23607816 | 4.95E-10 | 1.32E-07 |
| LSAT_5X72480 | 10.1635933 | -1.35656129 | 0.54210521 | 2.51E-05 | 0.00062295 |
| LSAT_6X41001 | 892.954178 | -1.35835486 | 0.16358651 | 1.15E-17 | 9.22E-14 |
| LSAT_5X97160 | 10.8028561 | -1.36028673 | 0.41780264 | 5.33E-06 | 0.00018822 |
| LSAT_5X161780 | 220.708398 | -1.36464298 | 0.33463273 | 5.27E-07 | 3.09E-05 |
| LSAT_4X124900 | 341.185899 | -1.37329223 | 0.1923359 | 8.37E-14 | 1.43E-10 |
| LSAT_2X68981 | 7.84099935 | -1.37754065 | 0.55213467 | 2.36E-05 | 0.00059725 |
| LSAT_8X15040 | 16.8002648 | -1.3812583 | 0.37458784 | 1.69E-06 | 7.83E-05 |
| LSAT_8X13440 | 288.237781 | -1.38268267 | 0.32122681 | 2.47E-07 | 1.75E-05 |
| LSAT_5X184481 | 42.9413625 | -1.3852303 | 0.2692084 | 8.74E-09 | 1.24E-06 |
| LSAT_6X10780 | 635.159681 | -1.38559619 | 0.37954696 | 1.86E-06 | 8.42E-05 |
| LSAT_8X51661 | 1142.56209 | -1.38807493 | 0.3503856 | 7.52E-07 | 4.12E-05 |
| LSAT_2X88041 | 50.0160766 | -1.40357911 | 0.38615494 | 1.90E-06 | 8.52E-05 |
| LSAT_8X52220 | 1219.95263 | -1.40407214 | 0.30988048 | 1.09E-07 | 9.07E-06 |
| LSAT_6X33120 | 56.5642861 | -1.4046989 | 0.27134739 | 7.62E-09 | 1.14E-06 |
| LSAT_1X60240 | 17.4828581 | -1.41286424 | 0.29947451 | 5.33E-08 | 5.04E-06 |
| LSAT_6X47280 | 179.398904 | -1.41707129 | 0.28297563 | 1.61E-08 | 2.01E-06 |
| LSAT_2X125601 | 43.0582261 | -1.4230273 | 0.33809162 | 3.33E-07 | 2.16E-05 |
| LSAT_2X127100 | 21.2166622 | -1.42365935 | 0.41299203 | 2.98E-06 | 0.00011965 |
| LSAT_1X78641 | 6.9344556 | -1.42887091 | 0.43518507 | 4.35E-06 | 0.00016072 |
| LSAT_6X13680 | 770.361721 | -1.43174552 | 0.64756137 | 2.80E-05 | 0.00067539 |
| LSAT_5X153021 | 180.864272 | -1.43296865 | 0.21836978 | 3.85E-12 | 2.64E-09 |
| LSAT_1X28480 | 327.419098 | -1.43457171 | 0.26045065 | 1.55E-09 | 3.27E-07 |
| LSAT_3X55860 | 6086.49009 | -1.4362856 | 0.51914409 | 1.22E-05 | 0.0003582 |
| LSAT_6X101780 | 933.564481 | -1.43820711 | 0.21896273 | 3.68E-12 | 2.64E-09 |
| LSAT_9X93180 | 120.509537 | -1.44219763 | 0.22713772 | 1.43E-11 | 6.62E-09 |
| LSAT_5X116940 | 78.8459656 | -1.44406567 | 0.18450211 | 4.83E-16 | 2.86E-12 |
| LSAT_2X19020 | 12.8016962 | -1.44534159 | 0.40097056 | 1.91E-06 | 8.55E-05 |
| LSAT_7X93960 | 1789.26354 | -1.46000536 | 0.2380063 | 5.08E-11 | 2.00E-08 |
| LSAT_8X158900 | 45.1380409 | -1.46699124 | 0.38498608 | 1.05E-06 | 5.43E-05 |
| LSAT_8X21421 | 17.2678403 | -1.48078666 | 0.37661257 | 7.24E-07 | 3.98E-05 |
| LSAT_6X113880 | 281.800939 | -1.48100843 | 0.38125526 | 8.29E-07 | 4.48E-05 |
| LSAT_6X34220 | 29.371923 | -1.4844307 | 0.41157883 | 1.74E-06 | 8.01E-05 |
| LSAT_9X108840 | 30.9895081 | -1.48906331 | 0.30414216 | 2.43E-08 | 2.69E-06 |
| LSAT_2X130341 | 1553.24877 | -1.49673026 | 0.21465764 | 2.28E-13 | 3.42E-10 |
| LSAT_3X30360 | 15.3113114 | -1.49780458 | 0.40141381 | 1.22E-06 | 6.03E-05 |
| LSAT_0X41520 | 8.49542611 | -1.51054744 | 0.41399687 | 1.46E-06 | 6.96E-05 |
| LSAT_1X81601 | 98.1216282 | -1.51542701 | 0.24086508 | 1.79E-11 | 7.98E-09 |
| LSAT_4X79301 | 86.2295967 | -1.51560045 | 0.29531481 | 8.35E-09 | 1.21E-06 |
| LSAT_5X88260 | 35.6374771 | -1.53177632 | 0.42869152 | 1.65E-06 | 7.74E-05 |
| LSAT_1X54920 | 306.097616 | -1.5342753 | 0.21100256 | 2.51E-14 | 5.02E-11 |
| LSAT_2X86961 | 60.3829496 | -1.53699907 | 0.27684651 | 1.06E-09 | 2.39E-07 |
| LSAT_1X111521 | 18.2110466 | -1.54642215 | 0.37167227 | 3.10E-07 | 2.05E-05 |
| LSAT_5X19740 | 34.4930673 | -1.55272274 | 0.28539859 | 1.78E-09 | 3.66E-07 |
| LSAT_6X42860 | 12.5459923 | -1.55478268 | 0.48317689 | 3.40E-06 | 0.00013327 |
| LSAT_4X62400 | 36.5959993 | -1.55541456 | 0.32366045 | 3.05E-08 | 3.22E-06 |
| LSAT_4X41620 | 31.121345 | -1.5708119 | 0.29010796 | 1.92E-09 | 3.91E-07 |
| LSAT_2X9220 | 88.0460352 | -1.57669706 | 0.31845677 | 1.57E-08 | 1.99E-06 |
| LSAT_5X78940 | 21.1699933 | -1.61112517 | 0.35874683 | 8.37E-08 | 7.22E-06 |
| LSAT_3X105460 | 14.0705111 | -1.62633459 | 0.33335179 | 1.78E-08 | 2.18E-06 |
| LSAT_9X3461 | 45.5811156 | -1.67642269 | 0.33060994 | 6.22E-09 | 9.82E-07 |
| LSAT_7X112821 | 40.6089619 | -1.6825292 | 0.33357806 | 6.78E-09 | 1.04E-06 |
| LSAT_2X96001 | 1950.74745 | -1.68415916 | 0.28244266 | 6.43E-11 | 2.34E-08 |
| LSAT_8X33320 | 44.1136918 | -1.68625779 | 0.44447787 | 5.12E-07 | 3.05E-05 |
| LSAT_8X37660 | 27.4759468 | -1.72449995 | 0.26431283 | 1.70E-12 | 1.75E-09 |
| LSAT_4X70021 | 57.0589453 | -1.75182762 | 0.35784022 | 8.53E-09 | 1.22E-06 |
| LSAT_8X71141 | 33.7531548 | -1.79584684 | 0.28103514 | 2.40E-12 | 2.06E-09 |
| LSAT_9X115461 | 240.836186 | -1.80835891 | 0.22023529 | 4.19E-18 | 5.02E-14 |
| LSAT_4X106840 | 21.0101399 | -1.81638962 | 0.43677143 | 8.39E-08 | 7.22E-06 |
| LSAT_3X99980 | 7.158218 | -2.30539071 | 1.85611148 | 1.91E-05 | 0.00051323 |

**Supplementary Table 2.** Overview of significant different expressed genes (DEGs) compared between lettuce leaves grown in chitin-amended potting soil and potting soil. The first column shows the locus tag of the corresponding gene in the genome of *Lactuca sativa* cv. ‘Salinas’. Column two to five show the base mean of the gene the log_2_-fold change of these gene in the chitin-treated condition, the standard error of the log_2_-fold change estimate (lfcSE), the *p*-value and the adjusted *p*-value.

| **Locus tag** | **Base Mean** | **log_2_-fold change** | **lfcSE** | ***p-*value** | ***p-*adj** |
| --- | --- | --- | --- | --- | --- |
| LSAT_2X4121 | 152.528268 | -1.68556232 | 0.41447253 | 2.55E-07 | 0.00056824 |
| LSAT_2X82160 | 120.418796 | -1.57317071 | 0.16438255 | 8.78E-23 | 1.76E-18 |
| LSAT_3X69900 | 86.6442074 | 1.21581503 | 0.44566804 | 4.31E-06 | 0.00345089 |
| LSAT_5X9120 | 110.436838 | 1.04848681 | 0.31178099 | 1.55E-06 | 0.00172617 |
| LSAT_5X98121 | 131.334677 | 1.08404151 | 0.84970788 | 2.81E-05 | 0.01281505 |
| LSAT_5X98101 | 169.290207 | 1.06970572 | 0.57220311 | 1.76E-05 | 0.00954372 |
| LSAT_5X106681 | 135.000594 | -1.73924709 | 0.53374076 | 9.75E-07 | 0.00123663 |
| LSAT_6X91781 | 1141.01653 | -1.17732782 | 0.32755654 | 8.70E-07 | 0.00123663 |
| LSAT_6X91761 | 1116.62759 | -1.44164212 | 0.49866453 | 2.61E-06 | 0.0023792 |
| LSAT_7X70721 | 52.340551 | -1.0771521 | 0.36398496 | 3.34E-06 | 0.00291282 |
| LSAT_8X74780 | 392.98167 | -1.79415481 | 0.73549479 | 2.11E-06 | 0.00201519 |
| LSAT_8X99301 | 104.079521 | -1.44156318 | 0.30683231 | 3.69E-08 | 0.00012334 |
| LSAT_8X159641 | 274.478332 | -1.48598195 | 0.21079196 | 8.34E-14 | 8.35E-10 |
| LSAT_9X7261 | 393.715567 | -1.03799685 | 0.41159121 | 7.25E-06 | 0.00514919 |
| LSAT_9X59600 | 329.929526 | -1.62007766 | 0.4787247 | 9.88E-07 | 0.00123663 |

**Supplementary Table 3.** Overview of the reference compounds for the targeted LC-MS metabolomics analysis.

| **Compound** |
| --- |
| Daidzin (internal standaard) |
| Pyrocatechol |
| Salicylic acid |
| 4-OH-Phenylacetic acid |
| Protocatechuic acid |
| Gentisic |
| *p*-Coumaric acid |
| *o*-Coumaric acid |
| Vanillic acid |
| Gallic acid |
| Caffeic acid |
| Hydrocaffeic acid |
| Quinic acid |
| Ferulic acid |
| Hydroferulic acid |
| Propyl gallate |
| Sinapinic acid |
| Resveratrol |
| 3,4,5-Trimethoxycinnamic acid |
| Galangin |
| Apigenin |
| Naringenin |
| Phloretin |
| Kaempferol |
| Luteolin |
| Aromadendrin |
| Epicatechin |
| Catechin |
| Quercetin |
| Hesperetin |
| Taxifolin |
| Cyanidin |
| Isorhamnetin |
| Chlorogenic acid |
| Apigetrin |
| Avicularin |
| Phloridzin |
| Cynaroside |
| Astragalin |
| Isoquercetin |
| Quercetrin |
| Chicoric acid |
| Quercetin-3-O-glucuronide |
| Procyanidin B2 |
| Naringin |
| Hesperidin |
| Rutin |

**Supplementary Table 4.** Reference genes tested for RT-qPCR. Genes selected were marked in bold.

| **Gene**  **symbol** | **Gene product description** | **Accession**  **number** | **Primers 5’-3’ (F/R)** |
| --- | --- | --- | --- |
| ACT | Actin | AY260165.1 | AGGGCAGTGTTTCCTAGTATTGTTG/  CTCTTTTGGATTGTGCCTCATCT |
| **APT1** | Adenosine phosphoribosyl transferase | AT1G27450 | CTGTACAAGAAGGAGAACGAGC/  ACGAGCACATACAGTGGCTT |
| EIF2A | Elongation initiation factor gamma subunit | EU028334 | TAGGCGAGTGGAGAAGCATT/  GTAGAAACAGCAACAGGCAAA |
| EIF4A1 | Eukaryotic translation initiation factor 4A1 | AT3G13920 | CTTCATAGGATTGGGCGAAG/  TATGAGATCCGCAACATTCG |
| PP2A | Serine/threonine protein phosphatase 2A | AT1G10430 | TTGACGGAATCGGAGGTAAAA/  CCGGCTGCACATTCCATT |
| **TIP41** | TAP42-interacting protein of 41 kDa | LACT_SATI.  CST1.6123 | GAGAGATTTGCTGGAGGGAAACTA/  CCTTTGACTGATGATGTTTGGA |
| UBC21 | Ubiquitin-protein ligase | AT5G25760 | TCTTAGATCACCGTCCCATCGT/  TCTGAGATTGTCCGAGGATATGAG |
| UBQ | Ubiquitin | GW397659 | AAGACCTACACCAAGCCCAA/  AAGTGAGCCCACACTTACGA |

**Supplementary Table 5**. List of primers tested for target genes. ^$^, primer sequences taken from Chalupowicz et al. (2021); *, primer sequences taken from De Cremer et al. (2013).

| **Gene**  **symbol** | **Gene description** | **Accession**  **number** | **Primers 5’-3’ (F/R)** |
| --- | --- | --- | --- |
| PR2^$^ | Pathogenesis related protein 2 | LSAT_3X7981 | TTGAGTGGATCCAACATTGAAG/  TCATGGATATTGGTCAAAGCAG |
| FLS2a^$^ | Flagellin-sensitive 2a | LSAT_4X152881 | ATTCCGGCGTCTATTTTCTGTA/  ATTAGTCAGCCACAAAGGGAAA |
| AOS^*^ | Allene oxide synthase | Lsa025821.1 | TCTTCCAACTCCAAAGTCGTCGCT/  TTGAGTGCATGGTGGGTAGGTTCA |
| MYB13^*^ | MYB-related transcription factor | Lsa042448.1 | TCTTCCCAAGCTCGCCGGTTTATT/  TCGTTATCTGTGCGACCCGGTAAT |
| PDR^*^ | Pleiotropic drug resistance protein 1-like | Lsa032832.1 | TTACAGAGAACGAGCTGCCGGAAT/  TCGAACCCAATCATCGCGTACACT |
| CLP30 | Calmodulin-like protein 30 | LSAT_2X84220 | AGGTGACCAAGGCGTTTCAA/  CACCTGTAACAGCTCCTCCG |
| STP | Sugar transporter ERD6-like 6 | LSAT_5X27561 | GACTTGCGGGTAGTGTAGCA/  TGTTTTCCCCTTGGTCTCCG |
| ARP | Auxin-responsive protein IAA14 | LSAT_5X66981 | AACGAGGCGGAGATGTTGAA/  CCAGCCTACCACTTGTGCTT |
| PAMPA70 | Pathogen-associated molecular patterns-induced protein A70 | LSAT_8X87760 | AAAATGTCCGTGCTCCGTCT/  CCGAGTTGAGCAGGTGAGTT |
| HLH137 | Transcription factor bHLH137 | LSAT_4X83840 | CTCCGGCCACCGATAACTAC/  TGCATGCATAAACTACATACACAT |
| TIP1-1 | Aquaporin TIP1-1 | LSAT_6X43221 | AGGGTGACGTCGGCATAATC/  GGGTCCAGCCCAGTAAATCC |
| PATL3 | Patellin-3 | LSAT_6X111521 | TCACCCAGAGGACCAAAAGC/  AGACCGCCGTATTGAACTGG |
| SAUR78 | Auxin-responsive protein SAUR78 | LSAT_8X85101 | TGCAGCAGAACTTGACCCAT/  ACAACCTCGCACTCAATCGT |
| GRP6 | Gibberellin-regulated protein 6 | LSAT_2X90361 | AACTGTCTCGGCAAAGGGAG/  CTGGAGGCACACACAGACAT |
| PR1^$^ | Pathogenesis related protein 1 | LSAT_5X111580 | TCGCCACAAGACTTTGTTAATG/  GAGGCAAGATTTTCACCATAGG |
| chaJ | Chalcone synthase J | LSAT_2X42860 | TCCGAGATCACCGCAGTTAC/  ATCGGGCAATGGGTCAGAAC |
| gluc18 | Beta-glucosidase 18 | LSAT_9X97401 | ACAAGGTGGATCAATCGGGA/  CCAGTACCCGGCCAATAGAG |
| FLR | Dihydroflavonol 4-reductase | LSAT_2X77261 | CATGTGGCTACGCCTATGGA/  TCCCCGCAGAGGATGTAAAC |
| Perox11 | Peroxidase 11 | LSAT_8X37660 | AGATTGCACAGCACACTCCA/  TAGCCTCAGTCGCAACCATT |
| Perox12 | Peroxidase 12 | LSAT_6X101780 | CGGTAAACCTTGTCCAGATGA/  ATGGATTCAAGCTGCGGACA |
| CHS | Chalcone synthase | LSAT_2X76880 | GACATTTGAGGGAGGTGGGG/  GGGCGATCCAGAAGATGGAG |
| MYB111 | Transcription factor MYB 111 | LSAT_9X181 | TCATCAAAGGTGCAACGGGA/  ATAGCTAGCGGCACTAGGGT |
| DFR | Dihydroflavonol 4-reductase | LSAT_2X77261 | TGACGAGTCTCATTGGAGCG/  GCTTGGAGGGAATGAGGGAG |
| BPS | (-)-Beta-pinene synthase | LSAT_6X32141 | ATTGGAGTGGGTGTAGTCGC/  GAACATCGCCTGTTTCCAGC |
| LCAT | Lecithin-cholesterol acyltransferase-like 1 | LSAT_9X81040 | CAAGAGCTTGGCTACACCGA/  GGTTGAGGAGTTGGAGGACG |
| CHS3 | Chalcone synthase 3 | LSAT_4X147220 | AGGGAGGCTGGACTGACTTT/  TAGCACATGTCTGCTAGCCC |
| CHILs | Chalcone isomerase-like protein 2 | LSAT_8X11480 | TTGTGTGTTCAGAAATGGTGATG/  GAGCGACGATCTCAGGATCA |
| ERF1^*^ | Ethylene-responsive element binding protein1 homlog | Lsa016859.1 | TCGCCGGTGATGTCCAGTTATCAA/  TGTTTCCCTCTCTGCTGGTTCACA |
| LOX^*^ | lipoxygenase | Lsa036946.1 | GCAACTAAGCGTGCTTCACCCAAT/  TGCCTCAAGAAGACCTCCACCATT |
| ARP14 | Auxin-responsive protein IAA14 | LSAT_3X125741 | CGGCTTTTTGAGTCAAGGGC/  GCGCTTCGACTTCTAGCCAT |
| GST | Glutathione S-transferase F11 | LSAT_3X87760 | CAAGTCCCGGTTGTTGAGGA/  AGTGATGCGCTTCGACTTCT |
| SABP | Salicylic acid-binding protein 2 | LSAT_3X91921 | AAGCCAACATCCTACCGATGG/  CTCCTCATCTCCGGTGGTTG |
| F3'H | Flavonoid 3'-monooxygenase | LSAT_5X23101 | ATCTACGCCTGCATCACTGG/  CGTCAACAAAGCCAAGTCGG |
| RLL1 | Transcription factor BHLH42 | LSAT_5X190001 | ACCAACCAGTCAACTTGGCA/  CGGCGCGATTTTTCGAATCT |
| ERF062 | Ethylene-responsive transcription factor ERF062 | LSAT_6X60300 | TAGAGGCGTAAGGCAAAGGC/  TAAAAACGCGCAATCGCCTC |
| G2bD | Gibberellin 2-beta-dioxygenase 1 | LSAT_9X101040 | GTCCACCCAATCCCTTCGG/  TCTTCACTGCTGTCACGTAAT |
| SGRL | Magnesium dechelatase SGRL | LSAT_6X97181 | TTTTAGGCCCTCCAGCAACC/  CCTCTCAGCTGGTCATGGTG |
| LCAT1 | Lecithin-cholesterol acyltransferase-like 1 | LSAT_9X81040 | GGGAGTGTGGTGGTCAATGT/  GCCAGCTCGTCCCCTTATAC |

# Supplementary figures


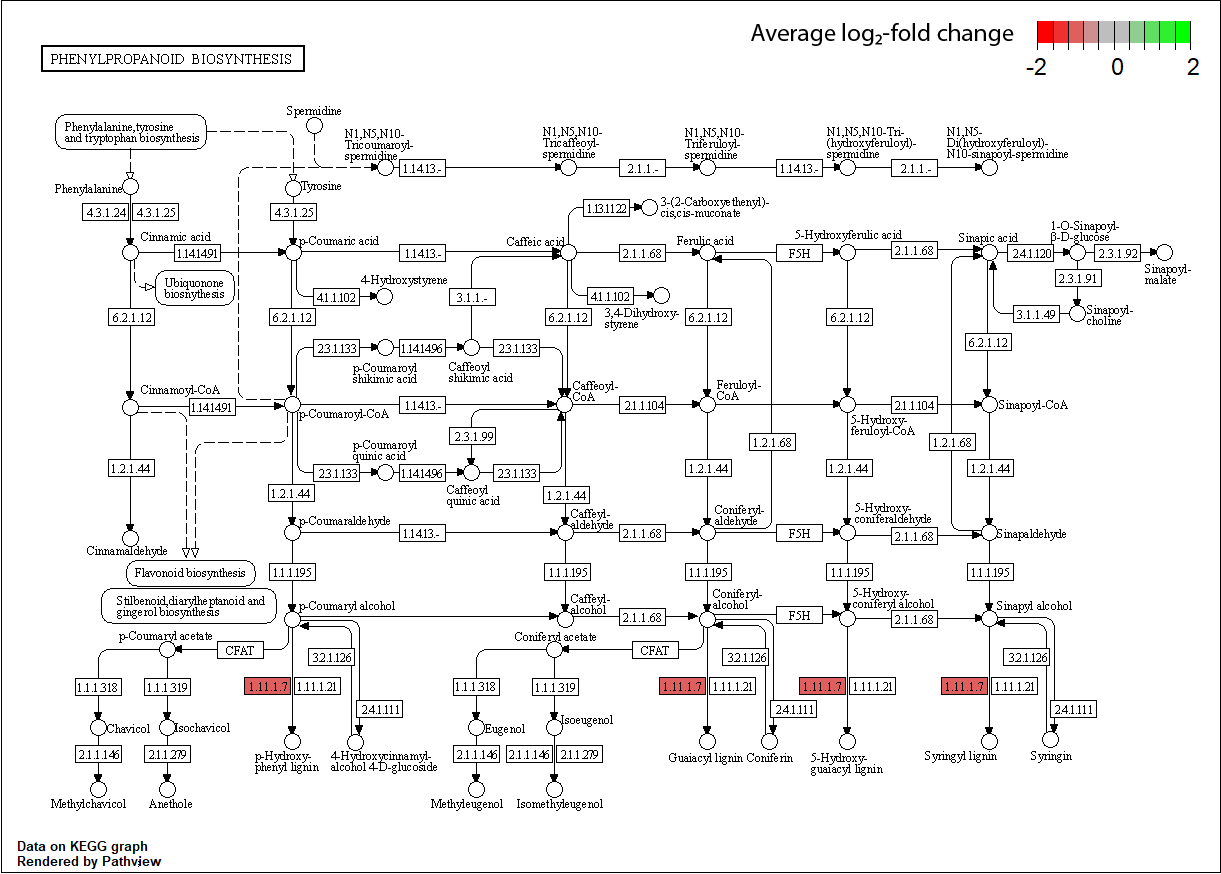


**Supplementary Figure 1.** KEGG pathway of the phenylpropanoid biosynthesis pathway with differentially expressed genes (DEGs) mapped to it. The pathway was downloaded from the KEGG database. The KEGG ortholog groups are colored according to the average log_2_-fold change of the different expressed genes mapped.


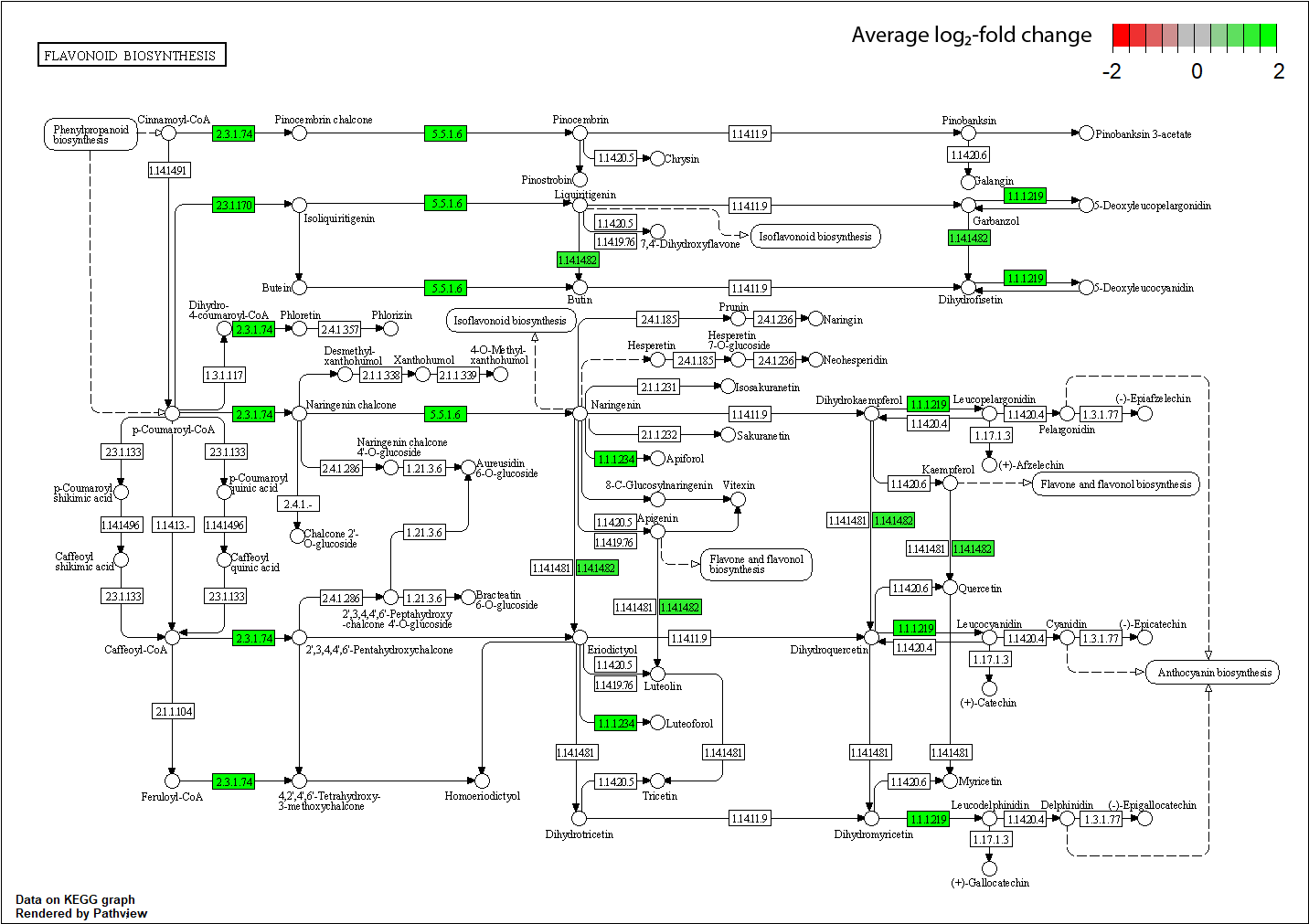


**Supplementary Figure 2.** KEGG pathway of the flavonoid biosynthesis pathway with differentially expressed genes mapped to it. The pathway was downloaded from the KEGG database. The KEGG ortholog groups are colored according to the average log_2_-fold change of the different expressed genes mapped.


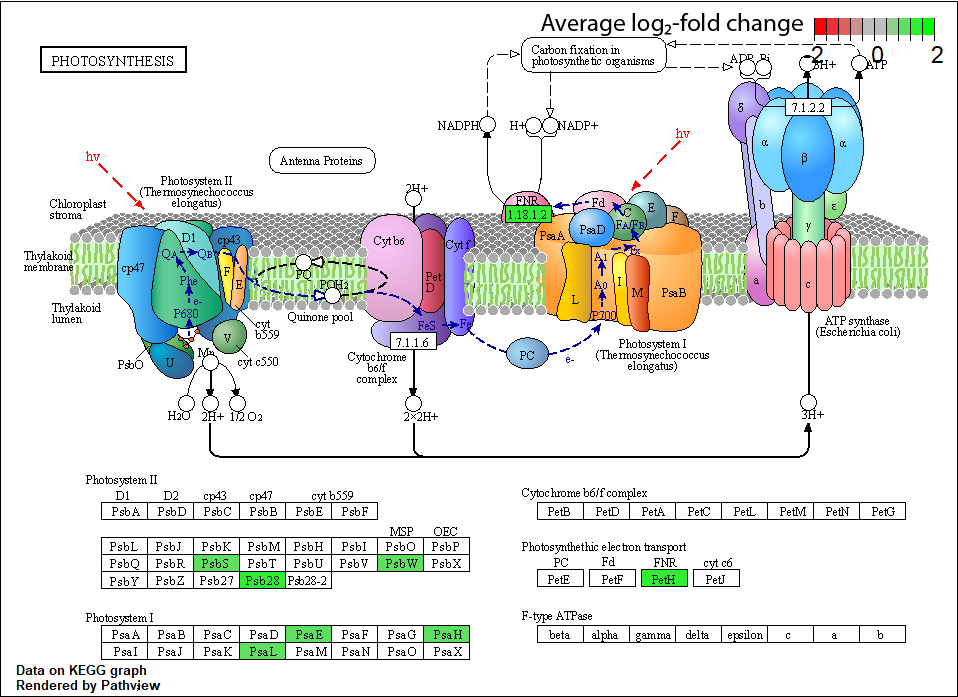


**Supplementary Figure 3.** KEGG pathway of the photosynthesis pathway with differentially expressed genes mapped to it. The pathway was downloaded from the KEGG database. The KEGG ortholog groups are colored according to the average log_2_-fold change of the different expressed genes mapped.

**
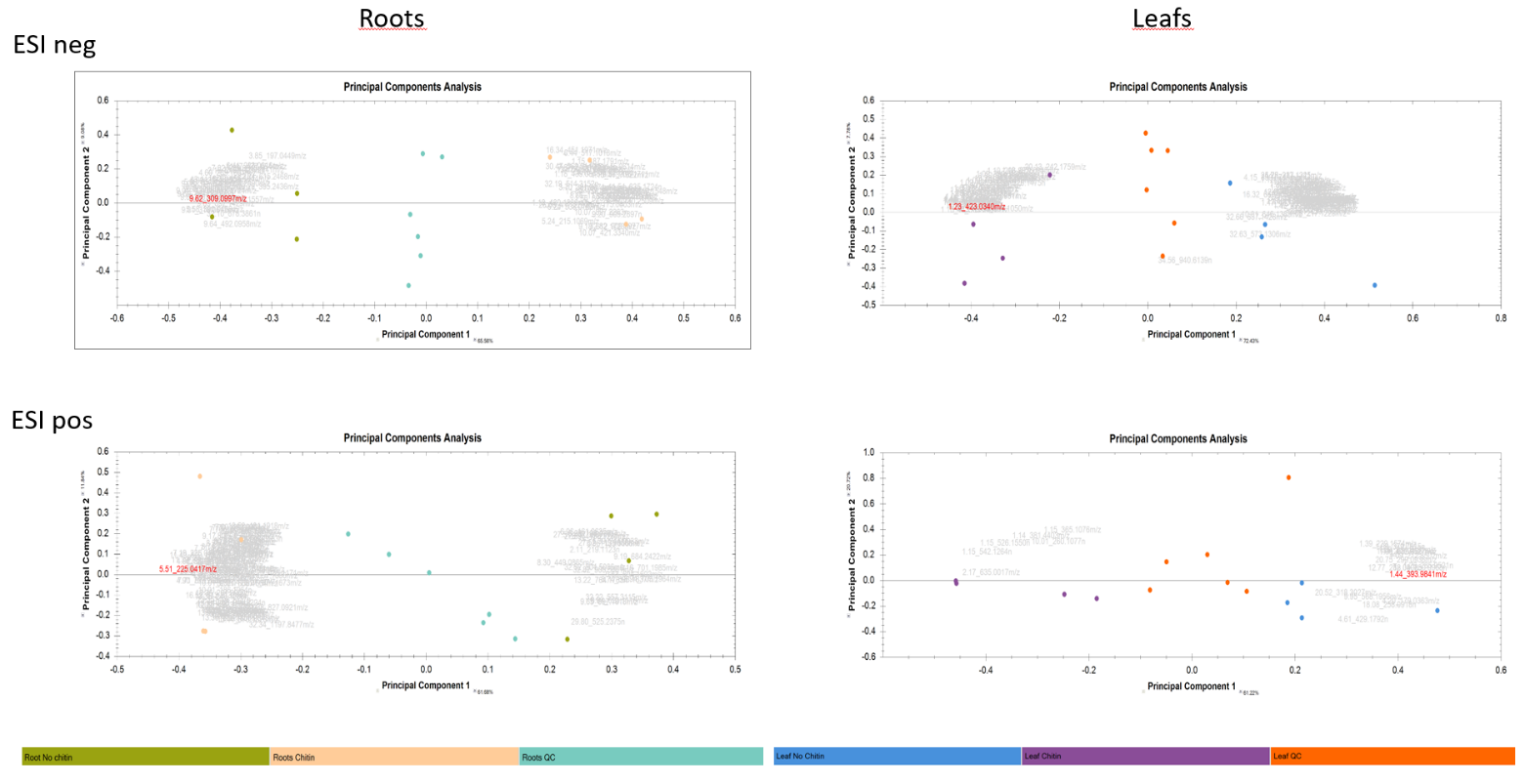
**

**Supplementary Figure 4.** Principal component analysis plot of untargeted method on the LC-HRMS in both positive ionization (ESI pos) and negative ionization (ESI neg). The grey cloud represents detected ions; the colored dots represent analyzed samples.


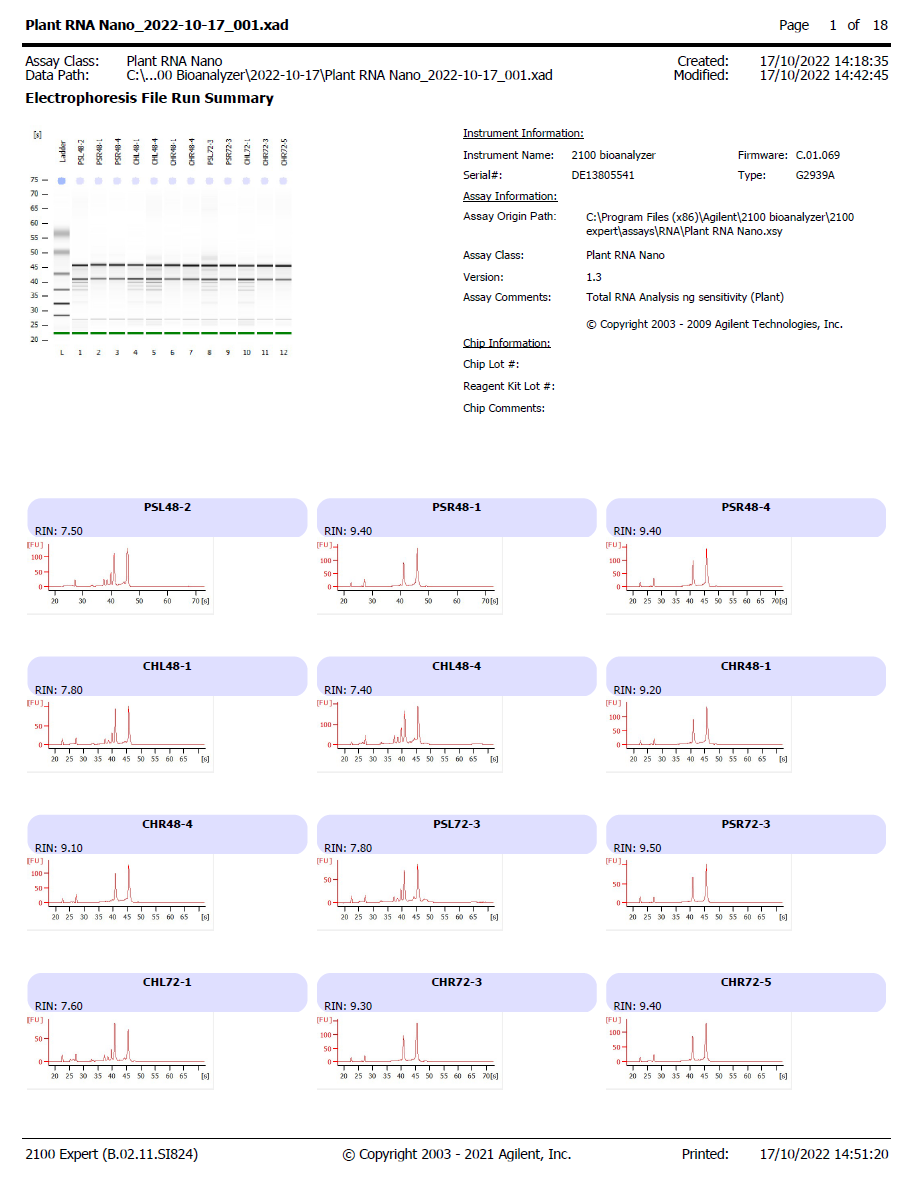


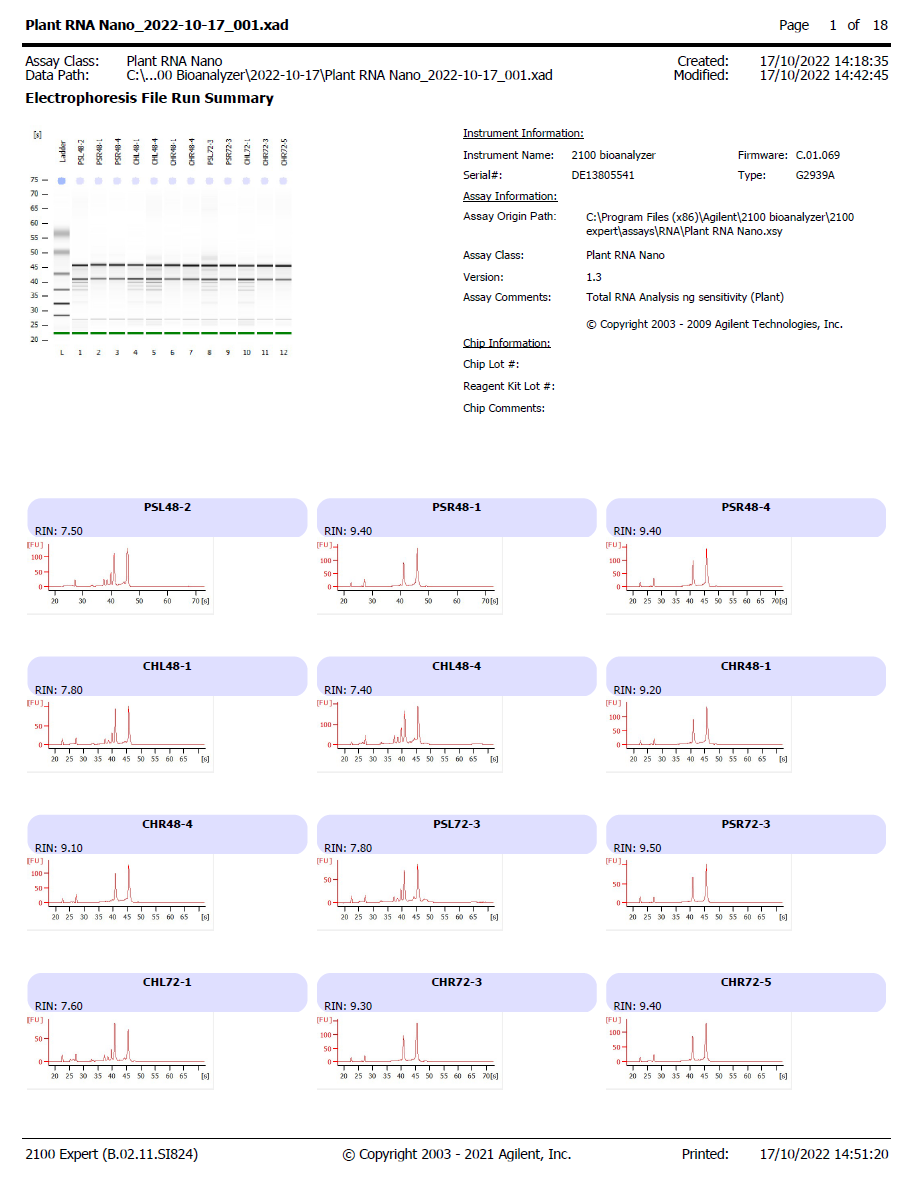


**Supplementary Figure 5.** Virtual gel electrophoresis and RNA integrity number evaluated using BioAnalyzer.


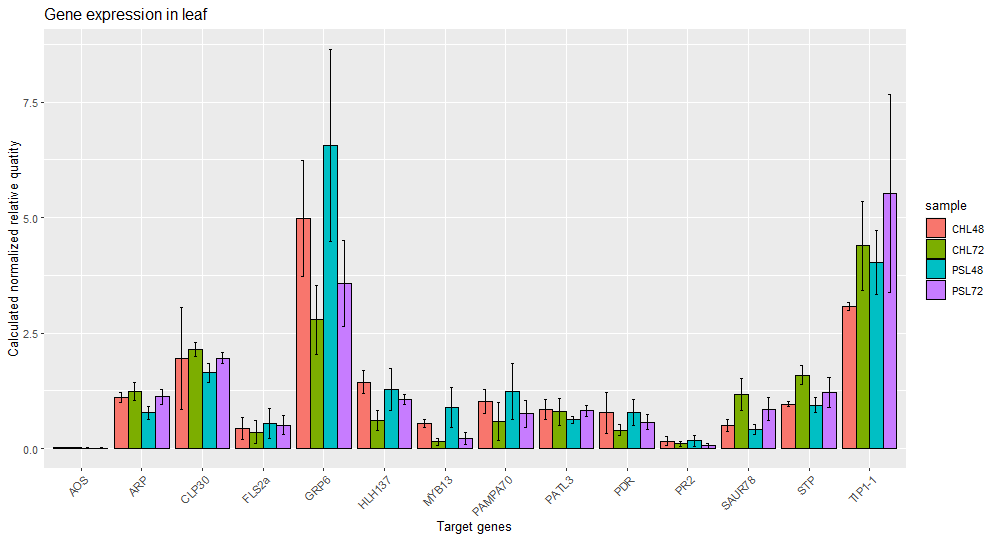


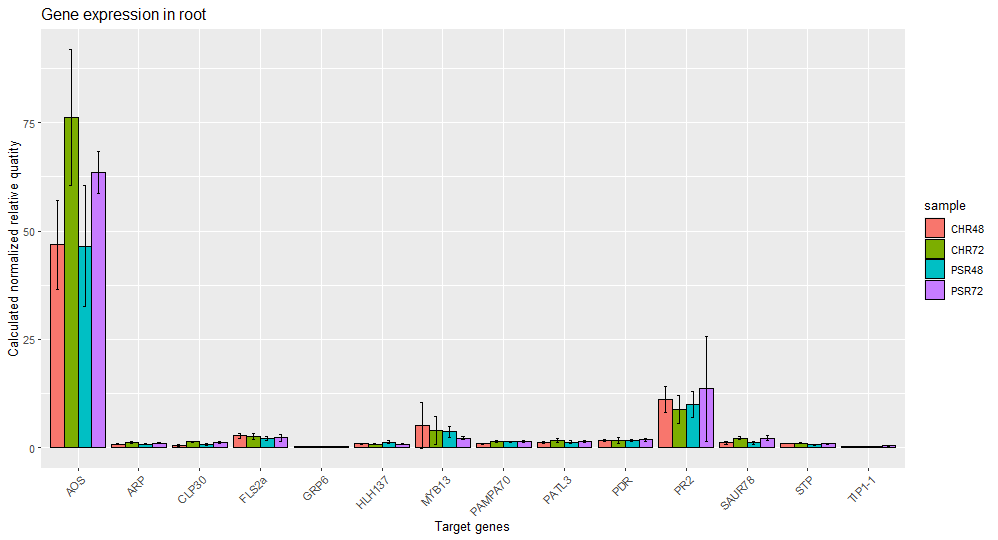


**Supplementary Figure 6.** Relative gene expression profiles in both leaf and root tissues obtained by RT-qPCR (calculated normalized relative quantity non-log-transformed). CHL/R48, chitin-treated leaf/root sampled at 48 hours post-transplanting (hpt); CHL/R72, chitin-treated leaf/root sampled at 72 hpt; PSL/R48, control leaf/root sampled at 48 hpt; PSL/R72, control leaf/root sampled at 72 hpt.

**References**

Borowski, J. M., Galli, V., Messias Rda, S., Perin, E. C., Buss, J. H., dos Anjos e Silva, S. D., and Rombaldi, C. V. (2014). Selection of candidate reference genes for real-time PCR studies in lettuce under abiotic stresses. *Planta* 239, 1187–1200. doi: [10.1007/s00425-014-2041-2](https://doi.org/10.1007/s00425-014-2041-2)

Chalupowicz, L., Manulis-Sasson, S., Barash, I., Elad, Y., Rav-David, D., and Brandl, M. T. (2021). Effect of plant systemic resistance elicited by biological and chemical inducers on the colonization of the lettuce and basil leaf apoplast by *Salmonella enterica*. *Appl. Environ. Microbiol.* 87, e0115121. doi: [10.1128/AEM.01151-21](https://doi.org/10.1128/AEM.01151-21)

De Cremer, K., Mathys, J., Vos, C., Froenicke, L., Michelmore, R. W., Cammue, B. P., and De Coninck, B. (2013). RNAseq-based transcriptome analysis of *Lactuca sativa* infected by the fungal necrotroph Botrytis cinerea. *Plant Cell Environ.* 36, 1992–2007. doi: [10.1111/pce.12106](https://doi.org/10.1111/pce.12106)

De Keyser, E., Desmet, L., Losschaert, M., and De Riek, J. (2020). A general protocol for accurate gene expression analysis in plants. *Methods Mol. Biol.* 2065, 105–118. doi: [10.1007/978-1-4939-9833-3_9](https://doi.org/10.1007/978-1-4939-9833-3_9)

Farmer, E. E., and Goossens, A. (2019). Jasmonates: what ALLENE OXIDE SYNTHASE does for plants. *J. Exp. Bot.* 70, 3373–3378. doi: [10.1093/jxb/erz254](https://doi.org/10.1093/jxb/erz254)

Luypaert, G., Witters, J., Van Huylenbroeck, J., De Clercq, P., De Riek, J., and De Keyser, E. (2017). Induced expression of selected plant defence related genes in pot azalea, *Rhododendron simsii* hybrid. *Euphytica* 213, 227. doi: [10.1007/s10681-017-2010-5](https://doi.org/10.1007/s10681-017-2010-5)

Sivasankar, S., Sheldrick, B., and Rothstein, S. J. (2000). Expression of allene oxide synthase determines defense gene activation in tomato. *Plant Physiol.* 122, 1335–1342. doi: [10.1104/pp.122.4.1335](https://doi.org/10.1104/pp.122.4.1335)

Untergasser, A., Nijveen, H., Rao, X., Bisseling, T., Geurts, R., and Leunissen, J. A. (2007). Primer3Plus, an enhanced web interface to Primer3. *Nucleic Acids Res.* 35(Web Server issue), W71–W74. doi: [10.1093/nar/gkm306](https://doi.org/10.1093/nar/gkm306)

Untergasser, A., Ruijter, J. M., Benes, V., and van den Hoff, M. J. B. (2021). Web-based LinRegPCR: application for the visualization and analysis of (RT)-qPCR amplification and melting data. *BMC Bioinformatics* 22, 398. doi: [10.1186/s12859-021-04306-1](https://doi.org/10.1186/s12859-021-04306-1)
